# Gut Microbiota-Immune System Interactions in Health and Neurodegenerative Diseases: Insights into Molecular Mechanisms and Therapeutic Applications

**DOI:** 10.14336/AD.2024.1362

**Published:** 2025-12-03

**Authors:** Rengasamy Balakrishnan, Shin-Il Kang, Ji-Yeon Lee, Yang-Kook Rho, Byoung-Kook Kim, Dong-Kug Choi

**Affiliations:** ^1^Department of Applied Life Sciences, Graduate School, BK21 Program, Konkuk University, Chungju, South Korea.; ^2^Department of Biotechnology, College of Biomedical and Health Science, Research Institute of Inflammatory Disease (RID), Konkuk University, Chungju, South Korea.; ^3^Department of Biotechnology, College of Biomedical and Health Science, Research Institute for Biomedical & Health Science (RIBHS), Konkuk University, Chungju, South Korea.; ^4^MEDIOGEN, Co., Ltd., Jecheon, South Korea.; ^5^PharmacoBio Co. Ltd., Seongnam, South Korea.

**Keywords:** Gut microbiota, gut-brain axis, immune system, gut dysbiosis, neurodegenerative diseases, microbiome-targeted therapies

## Abstract

The human body contains approximately 100 trillion microorganisms, predominantly within the gastrointestinal tract, collectively called the gut microbiota. Investigations have revealed the bidirectional communication between the gut microbiota and the brain, characterized as the "microbiota-gut-brain axis." This axis represents an important regulator of brain development and function, immune system development, and nutrient metabolism, making it a target for efforts to alleviate the development and progression of neurodegenerative diseases (NDDs). Despite extensive biomedical and clinical research, our understanding of the causes, optimal treatment, and progression of NDDs remains limited. This paper aims to summarize the available knowledge on the role played by gut microbiota and how it is connected to the progression of neurodegenerative conditions; in particular, the relationship between the microbiota and gut-brain communications and the gut microbiota and neuro-immune conditions is reviewed. We discuss how and why the gut immune system communicates with the brain and how this communication impacts neurodegeneration. Next, we examine the alterations in the gut microbiota, immune response, and brain changes associated with gut dysbiosis. Finally, we highlight the preclinical and clinical evidence for probiotics, prebiotics, fecal microbiota transplantation, dietary supplements, natural drugs, and exercise intervention as potential therapeutic approaches that could lead to a new treatment paradigm for NDDs.

## Introduction

Neurodegenerative diseases (NDDs) entail the gradual deterioration of specific neuronal populations, leading to motor and cognitive deficits. Common types include Alzheimer’s disease (AD), Parkinson’s disease (PD), Amyotrophic lateral sclerosis (ALS), Multiple sclerosis (MS), and Huntington’s disease (HD) [1, 2]. The onset and progression of NDDs are influenced by aging, genetic predispositions, and environmental factors, and emerging evidence suggests that peripheral factors also play crucial roles. The complex etiologies and origins of NDDs indicate that focusing exclusively on the end pathology of the central nervous system (CNS) may be insufficient to prevent the progress of NDDs [1].

The prevalence of AD among individuals aged over 85 years is 30%, with the annual incidence rate increasing from 0.5% in the 65-75 age group to 6%-8% in those over 85. In the case of PD, the incidence rate is 2% among individuals over 65 years. According to a 2023 report, the prevalence of ALS is approximately 9.1 cases per 100,000 individuals, while the global prevalence of HD is estimated to be 10.6-13.7 per 100,000 people. A 2019 report estimated that the prevalence of MS was 58 cases per 100,000 individuals in the population and has shown an increasing trend in the past two decades [3, 4], consistent with what one would expect in an aging population. Current conventional treatments have shown limited success in slowing down disease progression, but there is currently no effective therapy for curing or halting these diseases. Therefore, exploring and developing complementary approaches to establish more effective therapies are urgent objectives.

The gut microbiota refers to the trillions of unique microorganisms in the gastrointestinal tract, including archaea, viruses, fungi, and bacteria. These microorganisms play a crucial role in protecting against external pathogens and contribute to various physiological functions, such as the production of short-chain fatty acids (SCFAs), vitamin synthesis, breakdown of undigested carbohydrates, metabolism of essential substances (e.g., sterols, bile acids, and drugs), and hormone and neurotransmitter signaling [5]. Additionally, some gut microbes are involved in regulating the immune system. Gut colonization begins at birth and continues into the teenage years, developing a distinct intestinal human microbiota signature for each individual. Factors influencing the composition of an individual’s gut microbiota include the delivery method at birth, diet, geographical location, genetics, age, and sex [6]. Notably, the gene repertoire among the adult gut microbiota is estimated to contain 150 times more unique genes than the human genome. Furthermore, research has shown that the human gut microbiota markedly influences the composition of various positively upregulated and negatively downregulated plasma metabolites, potentially playing a more dominant role than genetics [7]. Notably, dysbiosis has been identified as one of the critical hallmarks of aging, further underscoring the microbiota’s crucial role [5].

The current corpus of empirical evidence has elucidated the bidirectional communication that exists between the CNS and the gut microbiota, commonly designated as the "microbiota-gut-brain axis." Notwithstanding the anatomical delineation between the gut and the brain, numerous pathways have been hypothesized through which the gut microbiota engages in communication with the CNS [8]. These pathways include the modulation of the immune response, circulatory system, neuroendocrine signaling, enteric nervous system (ENS), and vagal nerve activity facilitated by the production of neuroactive hormones, metabolites, and other substances. The gut microbiota possesses the capability to either produce or enhance the production of neurotransmitters such as dopamine, serotonin, and γ-aminobutyric acid (GABA) [9]. Previous investigations that highlighted the associations between the gut microbiota and CNS functionalities predominantly relied on simplified animal models, which proved to be inadequate for elucidating the fundamental mechanisms of action. Nevertheless, recent advancements in technological capabilities and culture-independent approaches have enabled researchers to move beyond simple correlative studies and engage in mechanistic investigations, thereby enlightening microbiota-host interactions [10]. Emerging evidence from both preclinical models and human studies has demonstrated that the gut microbiota modulates various aspects of behavior, including social interaction, physical performance, mental health, motivation, and cognitive functions such as memory. This complex interplay has led to the characterization of the gut microbiota as "the second brain"[11].

Furthermore, the gut microbiota’s role in NDDs is increasingly recognized. Early changes in the microbiota have been observed in preclinical AD and prodromal PD patients. Animal studies have provided compelling evidence that alterations in the gut microbiota contribute to NDD pathogenesis by influencing the host’s immune responses. Dysregulation of the immune system and immune signaling are key pathological features of NDDs. Changes in the gut microbiota and the production of microbial metabolites have been associated with various immune-related NDDs, including developmental disorders, neurodegenerative conditions, and emotional dysregulation. Recent research has also shown that the gut microbiota plays a vital role in developing and modulating host immune responses.

**Figure 1.**
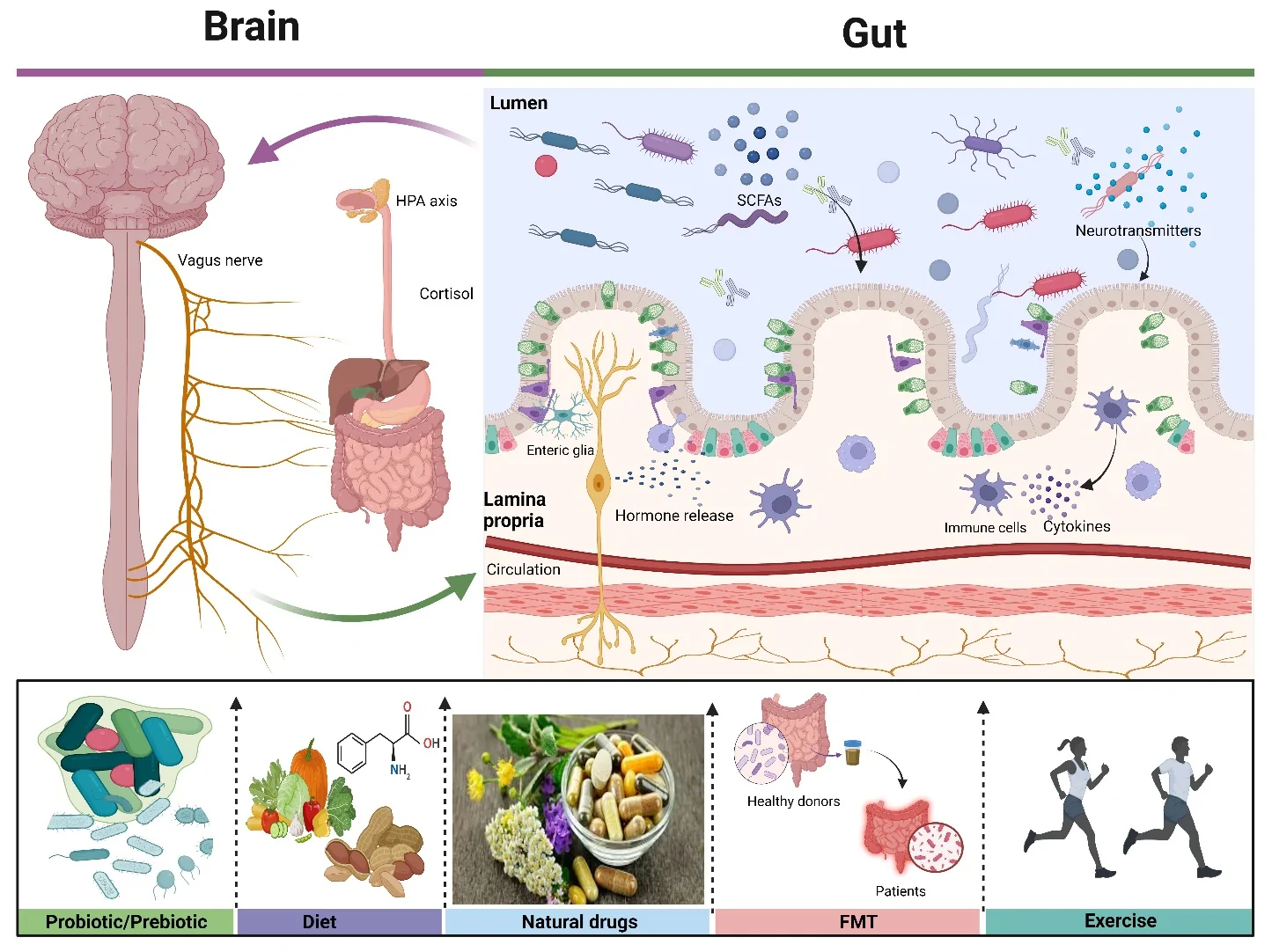
**Graphic abstract demonstrates that the microbiota-gut-brain axis has the potential to play a role in the development of NDDs and microbiota-targeting therapies for these conditions**. The pathways involved include the neural route, which includes structures such as the vagus nerve, the ENS, various neuroactive metabolites, and neurotransmitters, including butyrate. In addition, the immune route is represented by cytokines, while the neuroendocrine pathways involve gut hormone secretion, including neuropeptides and cortisol, particularly through the HPA axis. Neuroactive compounds, which include probiotics, prebiotics, dietary changes, natural substances, and metabolites produced by microbes, along with physical activity, impact the microbiome-gut-brain axis, influencing hormone release, gut barrier integrity, neurotransmitter synthesis, and enteric glial cells signaling, which are linked to the pathophysiology of NDDs. It was created with BioRender.com. ENS: enteric nervous system, HPA: hypothalamus pituitary adrenal, SCFAs: short-chain fatty acids, NDDs: neurodegenerative diseases.

In light of this context, the present review aims to explore the development of gut microbiota and its interplay with the human immune system. It will elucidate critical mechanisms that facilitate communication between the gut microbiota and the brain while also examining alterations in microbial composition associated with immune-related NDDs. Further, we highlight the preclinical and clinical evidence for probiotics, prebiotics, fecal microbiota transplantation, natural drugs, and exercise as potential therapeutic approaches that could lead to a new treatment paradigm for NDDs (Fig. 1).

### The role of the gut microbiota in brain development and function

The gastrointestinal tract represents a multifaceted organ system encompassing microorganisms, the mucosal immune system, and the intestinal epithelium. The assemblage of microorganisms—comprising viruses, fungi, archaea, and bacteria—living in the gastrointestinal tract is collectively called the gut microbiota [12, 13]. The gut microbiota exerts a dual impact on human health, manifesting both advantageous and detrimental effects. The microbiota provides benefits to the host by transforming dietary substrates into microbial metabolites, which facilitate inter-microbial communication and signaling interactions with host cells. This interaction further modulates the host's health status and disease susceptibility [14, 15]. Furthermore, the gut microbiota and its associated microbial metabolites are pivotal in maintaining gastrointestinal homeostasis and transmitting signals to distant organs, including the brain. Recently, it has become evident that the microbiota-gut-brain axis facilitates communication between the gut microbial community and the immune and hormonal systems that regulate brain function and development [16, 17].

Moreover, the developmental similarities between the gastrointestinal tract and the brain during the early stages of life are acknowledged [18]. The gut microbiota exhibits significant diversity and abundance in early life; disruptions during this pivotal period can adversely affect brain development and function [19]. For example, infants characterized by elevated levels of *Bacteroides* demonstrate higher cognitive outcomes, in contrast to those exhibiting high alpha diversity in gut microbiota, who tend to score lower on expressive language scales, visual reception, and overall composite [18]. Establishing gut microbiota during early life is essential for the development and maturation of both the immune and endocrine systems, exerting an impact on CNS function [16]. The absence of gut microbiota generally correlates with developmental challenges within the CNS involving neurotransmitter turnover and expression.

The blood-brain barrier (BBB) is established early in utero, constituting capillary endothelial cells sealed by tight junction proteins, pericytes, and astrocytes. Moreover, the gut microbiota may significantly impact the maturation of the CNS through its effects on the BBB, a selectively permeable barrier composed of endothelial cells that acts as a regulatory interface between the brain and systemic circulation, facilitating the selective exchange of molecules and nutrients essential for optimal brain maintenance and function [9]. A well-balanced gut microbiome and metabolites produced by microbes, such as SCFAs, play a crucial role in forming and maintaining a healthy BBB. The permeability of the BBB in developing sterile fetuses reduces as they reach adulthood [10]. In germ-free (GF) mice, the BBB exhibits increased permeability to macromolecules, attributed to the downregulation of key junctional proteins such as occludin and claudin-5 within the brain's endothelial cells. However, microbial colonization of the gut, along with the administration of butyrate—an essential SCFA derived from gut microbial fermentation—has been shown to reduce BBB permeability in GF mice [16].

Research involving GF animals and animals treated with broad-spectrum antibiotics is commonly conducted to explore the microbiota-gut-brain axis. These investigations aim to elucidate the developmental and behavioral implications arising from the total absence of gut microbiota. GF mice have been found to have deficits in brain function related to learning, recognition, and behavior [16, 20, 21]. These mice also exhibit altered levels of essential neurotransmitters—such as brain-derived neurotrophic factor (BDNF) and serotonin (5-HT)—in comparison to conventional mice [20, 22]. Moreover, exposing gut microbiota to GF mice during early developmental stages results in behavioral characteristics similar to those observed in specific pathogen-free mice. This suggests that early developmental periods may be pivotal for the gut microbiota's stimulus on neurodevelopment and behavioral outcomes [20]. Recent research has emphasized the impact of the gut microbiota on the development and functioning of microglia, the primary immune cells responsible for maintaining the CNS [23]. Studies involving GF mice and mice subjected to antibiotic treatment have demonstrated considerable deficiencies in microglial populations, characterized by diminished immature phenotypes and altered profiles of inflammatory cytokines that adversely affect the basal surveillance (M0) state [24, 25]. Other investigations involving GF mice have indicated increased BBB permeability, attributed to decreased expression of tight junction proteins occludin and claudin-5, potentially permitting harmful substances to infiltrate the brain, resulting in neuroinflammation and subsequent damage. Nevertheless, when GF adult mice were introduced to pathogen-free gut microbiota, BBB permeability was notably reduced, accompanied by an upregulation in the expression of tight junction proteins. [26]. These observations highlight the pivotal role of the gut microbiota in modulating neuroimmune and cognitive function.

### The microbiota-gut-brain axis

**Figure 2.**
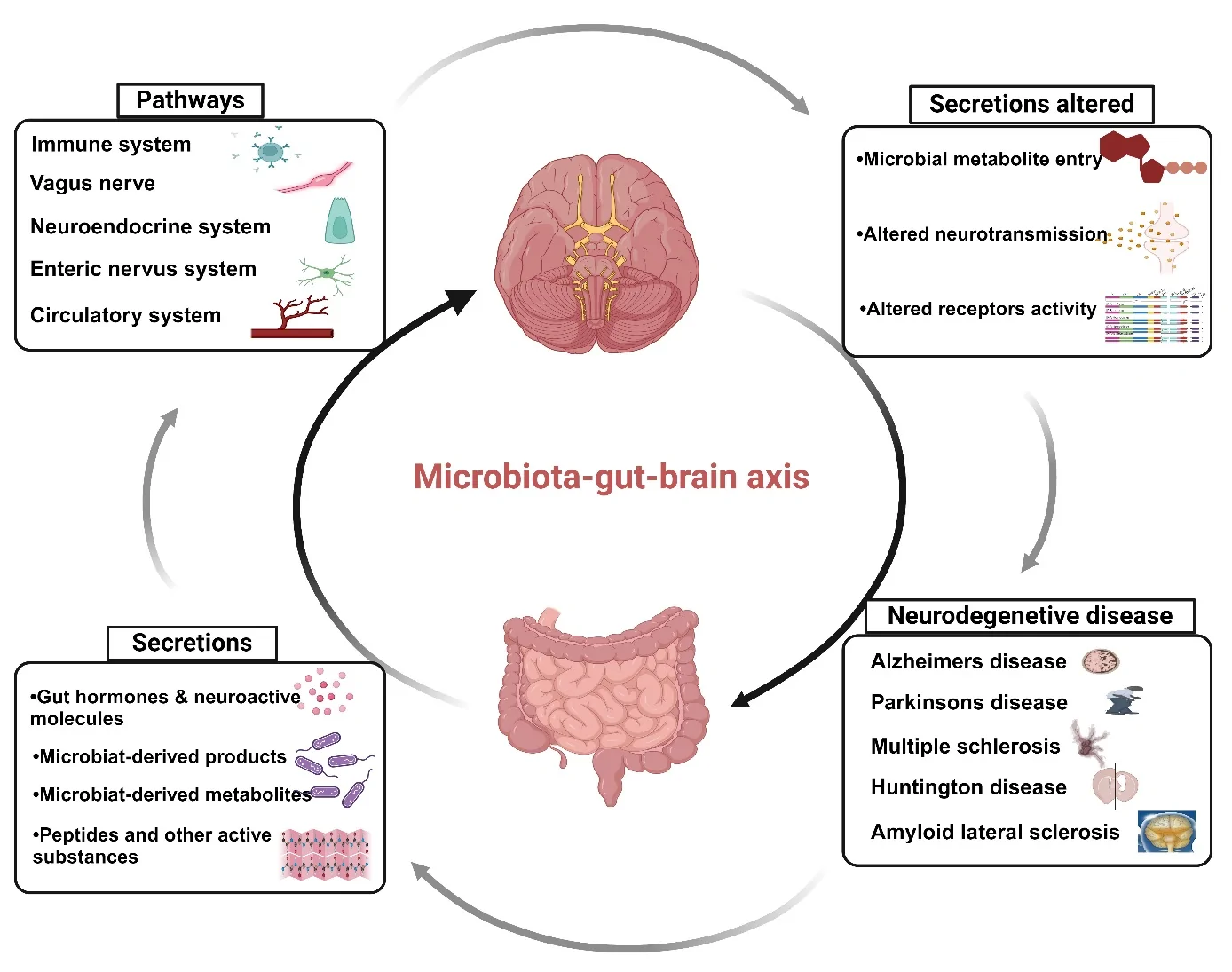
**The microbiota-gut-brain axis**. The vagus nerve, along with the immune, circulatory, enteric, and neuroendocrine systems, facilitates and the immune, circulatory, enteric, and neuroendocrine systems facilitate the bidirectional communication between the gut microbiome and the brain. Changes in gut microbiota composition have been associated with the onset of NDDs. It was created with BioRender.com.

The gut-brain axis is a critical communication system that regulates both gastrointestinal and brain function, and numerous pathways have been proposed to mediate communication within it. Signal passage along the gut-brain axis involves interactions among the ENS, CNS, autonomic nervous system (ANS), immune system, and endocrine system (Fig. 2) [27, 28]. The regulation of intestinal function and immune responses occurs at multiple levels, including systemic, regional, and local pathways. The ANS (comprising sympathetic and parasympathetic branches and driving both afferent and efferent signals), the HPA axis (through the systemic release of glucocorticoids), and the peripheral nervous system (PNS) (through the release of neuropeptides) play crucial roles in this regulation. Furthermore, neurally mediated responses are elicited by stimulating sensory afferents in reaction to variations in luminal composition or pressure, inflammatory processes, and muscular distension. Such responses may be conveyed to target cells via reflex arcs of local enteric nerves, thereby functioning independently of the CNS [29]. In addition to the extensively studied intestinal epithelial cells and resident immune populations, the microbiota also assumes a pivotal role in mediating brain-gut communications within the gastrointestinal system. This microbiota-gut-brain axis is fundamental to preserving gastrointestinal function and modulating cognitive and emotional behaviors [30, 31]. Disruptions within this intricate system have been associated with a variety of neurodevelopmental disorders as well as both gastrointestinal dysfunction and intestinal inflammation, including irritable bowel syndrome (Fig. 2) [30, 31].

The ANS constitutes a complex network that regulates the functions of the gastrointestinal tract, involving motility and mucus secretion. It facilitates the communication pathway between the gastrointestinal system and the CNS, thereby eliciting effects within the CNS that are pertinent to the processing of visceral sensory information. The ANS triggers direct neurological responses within the gut, leading to significant physiological alterations. Furthermore, it plays a pivotal role in mediating the interactions between gut microbiota and the ENS. The ENS activity, initiated by the ANS, results in the absorption and distribution of prebiotics and probiotics throughout the gastrointestinal tract, which includes complex carbohydrates and various microbial nutrients. Microorganisms possess the capacity to influence the neural system through neuromodulatory metabolites, which include GABA, catecholamines, serotonin, and tryptophan [32, 33]. Previous studies have shown how the microbiota interacts with the ENS. The gut microbiota—a major producer of serotonin in humans—is connected to the activation of the ENS through 5-HT receptors. De Vadder et al. [34] revealed this interaction through pharmacological manipulation of 5-HT receptors alongside the depletion of endogenous 5-HT. The application of a 5-HT receptor antagonist inhibits ENS activity. Additionally, the gut microbiota engages in communication with the HPA axis, which constitutes a principal neuroendocrine system that regulates the stress response. Signaling molecules produced within the HPA axis exert influence on the gut microbiota and are distributed throughout the organism. Recent studies using GF mice have found that there were enhanced plasma corticosterone concentrations, which suggests an overactive HPA axis and highlights the gut microbiota's significant impact [35, 36]. In human subjects, it has been observed that individuals afflicted with irritable bowel syndrome exhibiting alterations in gut microbiota typically demonstrate an exaggerated adrenocorticotropic hormone response to corticotropin-releasing factor infusion [37]. Although numerous studies have examined the bidirectional communication between the gut microbiota and the brain, achieving a comprehensive understanding of the underlying mechanisms continues to pose a considerable challenge.

**Figure 3.**
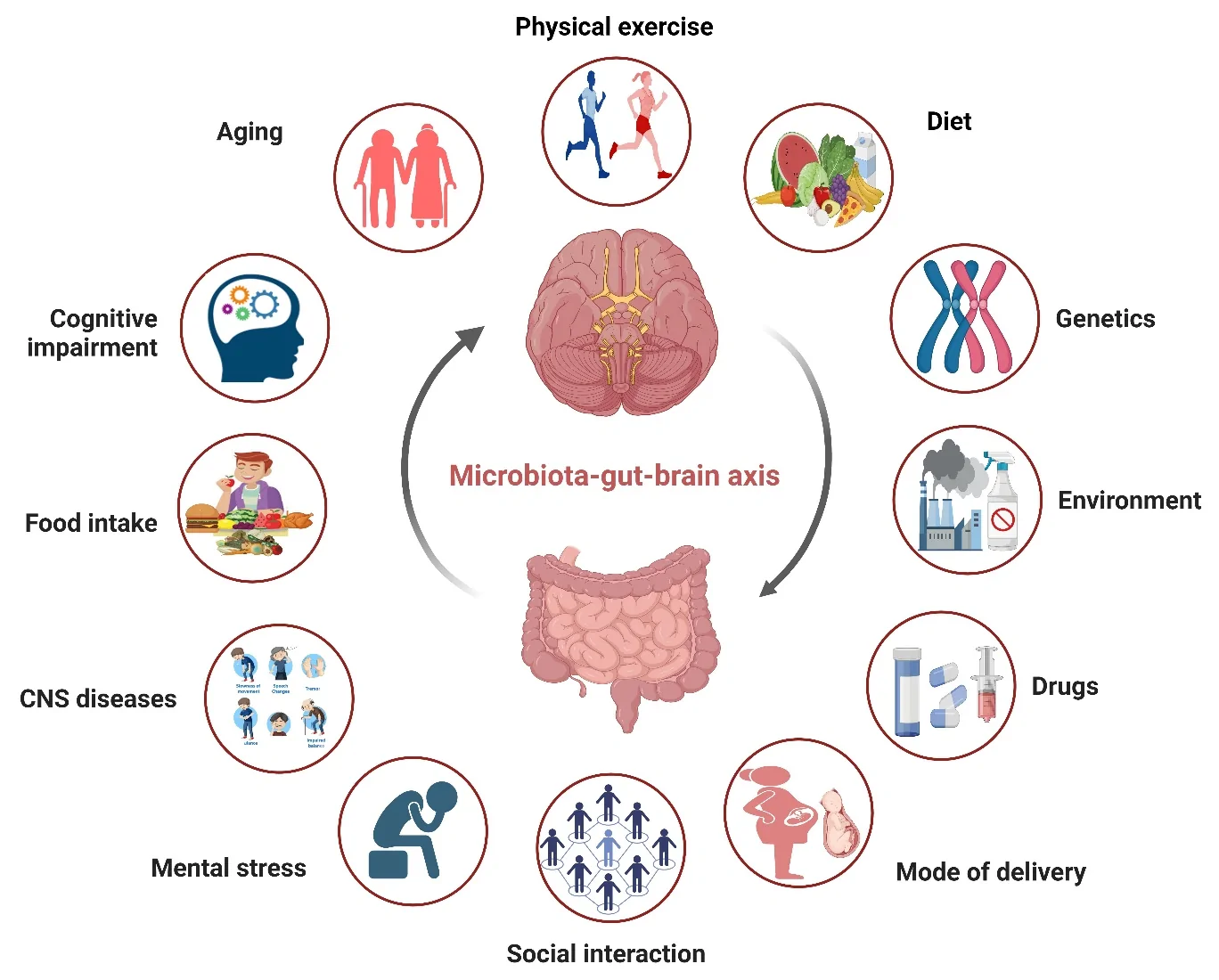
**The interactions between the bidirectional gut-brain axis and the common factors contribute to the gut-brain axis's activities**. It was created with BioRender.com.

Several intrinsic and extrinsic factors influence the gut-brain axis, including genetic predispositions, environmental conditions, socioeconomic factors, dietary habits, and pharmacological interventions (Fig. 3) [38]. Genetic and epigenetic influences are paramount in elucidating the intricate relationship between brain and gut health. Experimental investigations have established a correlation between the genetic composition of the host (whether human or animal) and the associated microbiota. A pivotal component of this interaction is the modulation of RNA. For instance, in GF mouse models, researchers identified dysregulation of microRNAs in specific cerebral regions, notably the amygdala (miR-182-5p and miR-183-5p) and prefrontal cortex (miR-219a-2-3p), thereby underscoring a significant connection between gut microbiota and neurological function [39]. Furthermore, an alternative investigation utilizing GF mice revealed that the gut microbiota exerted an influence on the expression levels of microRNAs and mRNAs within the hippocampus. Importantly, the reintroduction of gut microbiota in GF mice failed to reverse the documented behavioral alterations but did restore the levels of microRNAs and mRNAs [40].

Lifestyle changes, particularly dietary choices, can profoundly impact the gut-brain axis (Fig. 3). Studies have confirmed that a high-fat diet predominantly composed of animal-derived products can modify the composition of the microbiota. Animal research has indicated that a high-fat diet diminishes the levels of *Bacteroidetes* while augmenting those of *Proteobacteria* and *Firmicutes* [41, 42]. A notable abundance of *Proteobacteria* (specifically *Bilophila wadsworthia*) was observed in another study involving animals subjected to a high-fat dietary regimen. Conversely, a Mediterranean dietary pattern characterized by vegetables, nuts, whole grains, fruits, and select animal products (including fish and poultry) has demonstrated beneficial outcomes for hosts. Human interventional studies have indicated that adherence to the Mediterranean diet correlates with a reduction in the incidence of NDDs [43, 44]. Additionally, the ketogenic diet (KD) is associated with alterations in the microbiota composition; in particular, levels of *Akkermansia*, *Erysipelotrichaceae*, *Parabacteroides*, and *Sutterella* were observed to be elevated in mice administered KD [45]. Collectively, these studies substantiate the premise that lifestyle modifications significantly influence the gut microbiota.

Pharmacological agents, specifically antibiotics, exert a direct impact on the gut microbiota and, consequently, the gut-brain axis (Fig. 3). Several non-antibiotic drugs have also been implicated in the modification of gut microbiota composition, alongside neurophysiological and behavioral alterations [46]. The Belgian Flemish Gut Flora Project, an extensive investigation into the gut microbiota, identified various pharmacological agents—including antibiotics, hormones, osmotic laxatives, antihistamines, benzodiazepines, antidepressants, and medications designed for inflammatory bowel disease—as critically relevant to the variations observed in gut microbiota [47]. Additional studies have demonstrated that proton pump inhibitors, non-steroidal anti-inflammatory drugs, metformin, opioids, antipsychotics, statins, and thyroid hormones can likewise affect the gut microbiota [48]. Moreover, with the mounting interest in the gut-brain axis, it has been revealed that numerous psychotropic medications exhibit antimicrobial properties. For example, serotonin antagonists such as paroxetine, fluoxetine, and sertraline demonstrate antimicrobial efficacy against gram-positive bacterial strains, including *Staphylococcus* and *Enterococcus* [49]. These findings highlight the potential therapeutic impact of diverse pharmacological agents on the gut-brain axis.

### The immune system

The immune system plays a crucial role in the coordination of the gut microbiota-brain axis. The gut microbiota regulates immune cells present in both the gut and the brain [50]. Activation of the immune system in the gastrointestinal tract and the CNS is linked to responses to neuroinflammatory processes, worsening the pathophysiology associated with NDDs. Toll-like receptors (TLRs) on different types of immune cells commonly recognize microbe-associated molecular patterns (MAMPs), triggering their activation. Upon activation, immune cells secrete pro-inflammatory cytokines, which have the ability to cross the BBB and may play a role in the development and progression of NDDs [51]. In an experimental autoimmune encephalomyelitis (EAE) model, GF mice showed reduced production of pro-inflammatory cytokines, particularly interferon (IFN)-γ and interleukin-17A (IL-17A), in both the gut and spinal cord environments. Additionally, the introduction of segmented filamentous bacteria (SFB) in the host elicited T-helper 1 (Th1) and T-helper 17 (Th17) immune responses in the gut and spinal cord, exacerbating the symptoms of EAE in GF mice, as reported by Lee et al. [52]. Conversely, colonization by *Bacteroides fragilis* and *Prevotella histicola* mitigated EAE severity by enhancing the functionality of regulatory T-cells (Tregs), signifying that the gut microbiota exerts regulatory influences on neuroinflammation through immune-mediated mechanisms [53]. Additionally, SFB colonization triggered autism spectrum disorder-like symptoms through the modulation of Th17 cell populations in the intestine; however, the administration of neutralizing antibodies against IL-17A has the potential to alleviate behavioral deviations and abnormalities [54].

The activation of the inflammasome facilitates the maturation of caspase-1 and the subsequent secretion of pro-inflammatory cytokines IL-1β and IL-18, which play a significant role in neuroinflammatory processes. Specific MAMPs have the capacity to initiate inflammasome signaling pathways, which results in the production of pro-inflammatory cytokines that are implicated in various NDDs [55]. Furthermore, mice models exhibiting a genetic deficiency in caspase-1 demonstrate a reduction in behaviors indicative of depression and anxiety in response to chronic stress [56]. Additionally, the gut microbiome directly influences the functionality of immune cells residing in the CNS. Molecules originating from the gut microbiota, which possess the ability to traverse the BBB, may significantly affect the development and activation of brain-resident immune cells, including microglia and astrocytes [50]. Microglia within the CNS are integral to brain development, maintenance of homeostasis, and the progression of disease. Similar to other macrophages that inhabit tissues, microglia execute their roles in the CNS through the release of cytokines, activation of the complement system, and phagocytic activity [57]. Microglia in murine models with depleted gut microbiota demonstrate alterations in the expression of genes associated with inflammation and exhibit characteristics of immaturity [24]. However, the underlying mechanisms through which gut microbiota exert influence over microglia remain inadequately elucidated. In addition to microglia, astrocytes represent essential immune cells among glial cells, engaged in a myriad of functions, including the regulation of the BBB, the modulation of CNS development and repair via the release of cytokines and chemokines, and the presentation of antigens. The signaling of type 1 IFN in astrocytes, mediated by microbial tryptophan metabolites and the activation of aryl hydrocarbon receptors (AHRs), may contribute to the attenuation of CNS inflammation [58, 59]. Consequently, the management of immune cell equilibrium may provide a viable strategy for modulating communication within the gut-brain axis.

### Microbiota and the gut: gut-immune system interaction

**Figure 4.**
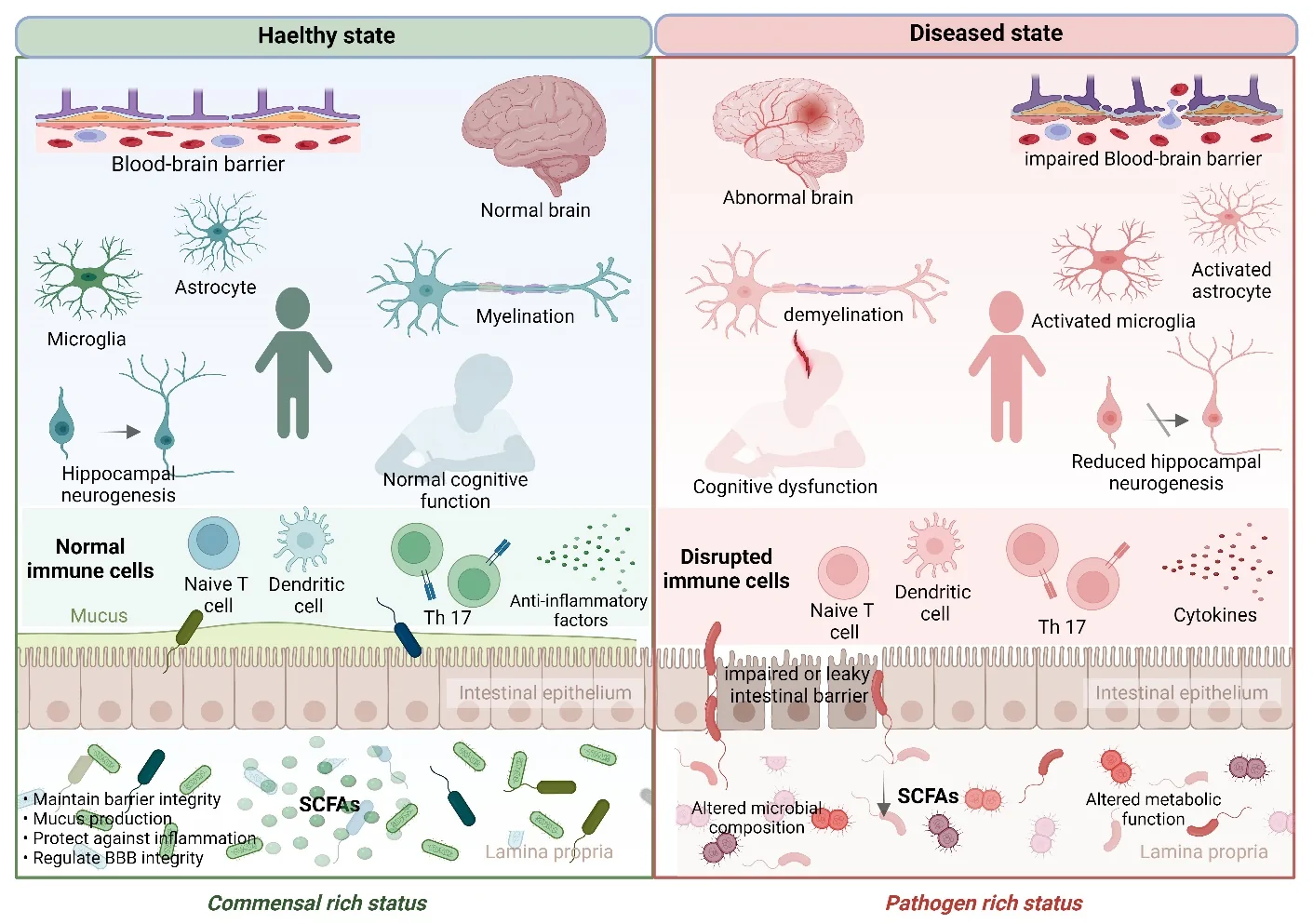
**The involvement of microbiota-related chronic neuroinflammation in both healthy and diseased conditions is an important factor to consider**. It was created with BioRender.com.

Recent research indicates the existence of a communication network between the gut microbiota and the immune system. This network plays a crucial role in the immune system’s early postnatal development and continues to regulate the immune system and its response to self-antigens across the host’s lifespan. The “gut-brain axis” is a recently identified concept that involves the neuro-entero-endocrine system, a modulated endocrine system at the intestinal level. This system interacts with the immune system at the mucosal level to maintain homeostasis and improve protection against microbial invasion in pathological conditions. Therefore, changes in microbiota composition may be linked to various CNS disorders, including neuropsychiatric, neurodegenerative, and neuro-inflammatory disorders (Fig. 4) [54].

The gut plays a crucial role in supporting the immune system, as approximately 70% of the immune system is located there. The gut contains numerous microorganisms, and their interactions with the host are vital for developing and maintaining the host’s immunity [60, 61]. The immune system comprises two main components: the adaptive immune system and the innate immune system. The innate immune system offers nonspecific defense through a variety of protective mechanisms, including chemical defenses (e.g., the enzymatic substances and antimicrobial peptides), physical barriers (such as skin and mucosal surfaces), and innate immune cells (including macrophages, granulocytes, and natural killer cells) [62]. Conversely, the adaptive immune system is characterized by the involvement of T and B lymphocytes, which possess the capability to identify and react to particular foreign antigens. T cells are responsible for recognizing pathogens that have infiltrated host cells, and this specific form of adaptive immunity is termed cellular immunity. Furthermore, T cells are involved in modulating the activity of B cells, which are responsible for the synthesis of antibodies and proteins that can specifically identify antigens. The protection provided by B cells through the circulation of antibodies in bodily fluids is referred to as humoral immunity [63]. This interconnected network of cells plays a crucial role in maintaining and restoring tissue function when exposed to microbes and environmental factors (Fig. 4). This balance, known as homeostasis, is essential for the proper development of the immune system, which relies on the presence of beneficial microorganisms [64]. Studies involving GF mice and animals treated with antibiotics have shown that the immune system’s maturation is closely linked to establishing a diverse microbiota [65]. GF mice display underdeveloped gut-associated lymphoid tissues (GALT), a reduced intestinal lymphocyte count, diminished production of immunoglobulin (Ig) A, and impaired antimicrobial peptide function compared to their colonized counterparts [66]. While many of these deficiencies can be reversed by introducing a microbiota, some components can be fully restored only if colonization occurs early in development [67].

The immune system initially encounters commensal microorganisms during parturition as the neonate traverses the birth canal, and subsequently through maternal lactation (although there exists some contentious evidence positing the potential for microbial exposure in utero, this is presumably restricted to microbial byproducts or bacterial DNA, as viable microbes are absent in that environment and placental samples are likely subject to contamination during the birthing process) [68, 69]. These formative interactions are essential for the maturation of both the mucosal and systemic immune systems into adulthood [69]. The capacity to establish immune tolerance at an early developmental stage may be attributable to the underdeveloped state of the neonatal immune system coupled with the tolerogenic milieu prevalent during this phase. In the initial stages of life, the immune system exhibits a reduced production of inflammatory cytokines, thereby facilitating a predominance of regulatory T and B cell differentiation [70]. TLRs, which serve as innate immune pattern recognition receptors, are integral to the normative development of the intestinal mucosal immune system and are adept at recognizing microbial pathogens pertinent to infants, including group *B Streptococcus*, *Listeria monocytogenes*, and respiratory syncytial virus (RSV) [68]. Mononuclear cells derived from infants demonstrate diminished expression of specific cytokines when juxtaposed with those sourced from adults, with this expression exhibiting an upward trajectory between birth and 1-2 years of age. Moreover, TLR activation by antigens derived from the typical intestinal microbiota conveys signals that inhibit inflammatory processes and support the maintenance of intestinal homeostasis [68]. Nucleotide-oligomerization-domain (Nod)-like receptors (NLRs) are capable of discerning particular microbial components and instigating the assembly of inflammasomes, functioning as sensors for MAMPs. A deficiency in NLRs is correlated with aberrant immune responses and cognitive impairments [71, 72]. The establishment of microbiota within the gastrointestinal tract during early development significantly influences the maturation of T cell populations, encompassing T helper cells (Th1, Th2, and Th17) as well as regulatory T cells (Tregs) [73]. For instance, GF mice exhibit diminished induction of Treg cells, a complete absence of Th17 cells, and an imbalance in the Th1/Th2 ratio that favors Th2 responses [74]. Colonization with *Bacteroides fragilis* restores the development of the Th1-associated immune response via a pathway dependent on the bacterial product polysaccharide A [75]. Furthermore, *Bacteroides fragilis* promotes the accumulation of Tregs through TLR-2 activation while concurrently inhibiting the development of Th17 cells [76].

The gut microbiota substantially affects antibody responses, but they can still occur in animals lacking it [77]. IgA secretion by intestinal plasma cells is rapidly initiated following microbial colonization and can occur via both T-dependent and T-independent pathways. This process leads to the production of polyreactive IgA with a low affinity for commensal bacteria, which supports the coating of surface microbes. Once produced, IgA is transported across the epithelial barrier and secreted into the intestinal lumen. Research has shown that IgA-mediated coating can enhance the intestinal colonization of certain commensal strains [79]. The microbiota can influence antibody production by producing ATP through a mechanism that involves purinergic receptor P2X7 (P2X7)-mediated signaling of T follicular helper cells, restricting the production of protective IgA against enteropathogens [80].

### The interaction between the gut immune system and the brain

The gut-brain axis enables bidirectional communication through hormonal, neural, and immune pathways, which provide a physical and chemical connection through the functional lymphatic vasculature and the BBB. Changes in one organ can impact another because of signals between the two. For example, certain bacteria can produce hormone-like chemicals and monoamine neurotransmitters in the gut, directly influencing the gut’s immune system and brain. Several findings indicate that gut microbes also activate peripheral immune cells, which regulate responses to brain injury, autoimmunity, neuroinflammation, and neurogenesis. As a result, both the immune system and gut microbiota contribute to regulating neurodevelopmental and psychiatric diseases and NDDs, suggesting an important role for immune cells in brain homeostasis and during microbial and environmental challenges (Fig. 4) [81]. The oligodendrocytes, microglia, and astrocytes play a pivotal role in regulating the immune system within the CNS. This phenomenon occurs due to the BBB inhibiting circulating T and B lymphocyte entry into the underlying parenchyma. Empirical studies have demonstrated that the gut microbiota can influence and regulate microglia's maturation and functional properties, thereby underscoring its essential significance within the CNS [24, 26].

### Microglia

Microglia, recognized as the resident macrophages, play a pivotal role in the maintenance of homeostasis, the regulation of synaptic transmission, the process of synaptic pruning, and the formation of neuronal circuits [82]. They engage in protective mechanisms against a variety of pathological states through the activation of immune responses, phagocytic activity, and the synthesis of cytokines (Fig. 4) [83]. In GF mice, there is a notable increase in the prevalence of immature microglia across various brain regions—including the hippocampus, cerebellum, and cortex—when compared to controls that are specific pathogen-free (SPF)-colonized [22]. These microglia exhibit a more branched phenotype and reduced expression of genes associated with maturation to an active phenotype, indicating their immaturity. Similarly, antibiotic treatment in colonized wild-type (WT) mice has been linked to an increased proportion of immature microglia, although total microglia numbers remained unchanged, and a normal phenotype was restored following the reintroduction of a complex microbiota [24]. These findings reveal the microbiota’s role in microglia development, which depends on the developmental timing and/or duration of microbial colonization.

Although the pathways by which gut microbiota modulate brain microglia are not fully elucidated, it appears that these effects are more significantly influenced by the diversity and complexity of the bacterial community rather than by the presence of specific bacterial taxa. For example, GF mice that are colonized with a limited consortium of bacterial species (including *Bacteroides distasonis*, *Lactobacillus salivarius*, and a member of *Clostridium cluster* XIV) still presented with microglial abnormalities [84]. Conversely, the oral administration of a comprising the three principal SCFAs—acetate, propionate, and butyrate—facilitated the maturation of microglia. SCFAs possess the ability to penetrate the BBB and may exert direct effects on microglial cells [85]. Despite the limited understanding of how they work, SCFAs can act as signaling molecules by interacting with G protein-coupled receptors (Gpr41 and Gpr43), which are widely found in intestinal epithelial and immune cells [86]. The activation of these receptors can lead to an inflammatory immune response, increase gastrointestinal permeability, and promote the absorption of neuroactive metabolites [87]. Additionally, SCFAs can directly stimulate the sympathetic nervous system through Gpr41, which is present in sympathetic ganglionic neurons [88], thus affecting catecholaminergic systems by regulating the expression of the tyrosine hydroxylase gene [89]. Nevertheless, the immature microglia from GF mice cannot effectively respond to stimuli and show diminished responses when exposed to LPS or lymphocytic choriomeningitis virus, resulting in shape changes and reduced expression of several genes related to interferon responses, pro-inflammatory cytokines, and effector processes. These effects indicate that immune-stimulating substances from the gut affect the function of microglia, potentially protecting the CNS from damage during viral or bacterial infections [90].

### Astrocytes

Astrocytes, which represent the most abundant group of glial cells within the CNS, are integral to the preservation of both the structural integrity and functional equilibrium of the CNS, encompassing tasks such as the maintenance of the BBB, the regulation of ionic gradients, the modulation of neurotransmitter concentrations, and the facilitation of nutrient transport. Moreover, these cells possess the capability to assimilate information from adjacent glial, neuronal, vascular, and immunological entities, thereby influencing neural excitability and the processes of synaptic formation [91]. Although traditionally not classified as immune cells residing within the CNS, astrocytes exhibit specific characteristics, including the expression of MAMPs and major histocompatibility complex class II antigen-presenting receptors, enabling them to detect danger signals and respond by secreting cytokines and chemokines, as well as activating adaptive immune mechanisms (Fig. 4) [92].

The gut microbiota possesses the potential to modulate the functional activity of astrocytes through microbial metabolites that activate aryl hydrocarbon receptors (AHRs) [93]. AHR functions as a ligand-dependent transcription factor that synthesizes signals derived from environmental, microbial, dietary, and metabolic origins to coordinate intricate transcriptional activities [94]. Metabolites generated by the gut microbiota can bind to AHR, thereby inducing anti-inflammatory responses by restricting the recruitment of neurotoxic immune cells through specific signaling pathways. Investigations involving mice subjected to ampicillin treatment have demonstrated that diminished AHR activation correlates with exacerbated disease symptoms in the absence of AHR activation. Bacteria susceptible to ampicillin are capable of converting dietary tryptophan into AHR agonists; consequently, mice receiving ampicillin exhibited reduced AHR activation and augmented severity of symptoms in EAE, a murine model of MS. However, supplementation with tryptophan in EAE mice resulted in a reduction of pro-inflammatory molecules produced by astrocytes and alleviated symptom severity [95, 96].

Furthermore, metabolites produced from tryptophan by the microbiota can impact the activation of astrocytes through microglial cells. In the EAE mouse model, microglial cells, activated by dietary tryptophan metabolites and mediated through AHR, controlled astrocytes' pro-inflammatory and neurotoxic functions by generating TGF-α and VEGF-B. The TGF-α derived from microglia counteracts pathogenic activation through the epidermal growth factor receptor (ErbB-1) receptor, while microglial vascular endothelial growth factor-B (VEGF-B) triggers Fms-related receptor tyrosine kinase 1 (FLT-1) signaling in astrocytes, worsening EAE by promoting increased demyelination and the recruitment of CNS monocytes [97]. In human studies, astrocytes exposed to tryptophan metabolites such as indole-3-aldehyde and indole-3-propionic acid showed reduced expression of pro-inflammatory markers (e.g., CC chemokine family 2 (Ccl2), IL-12, IL-6, IL-23a, and nitric oxide synthase 2 (Nos2)), which are known as biomarkers associated with MS in humans [97]. These findings suggest that dietary tryptophan's microbial metabolites could regulate astrocytes' inflammatory state, thereby contributing to neuroinflammation.

### Changes in the gut environment, gut immune system, and brain during dysbiosis

The dysregulation of the immune system, resulting from an imbalance in gut microbiota, results in a compromised intestinal barrier and sustained inflammation, activating many processes associated with neurodegeneration [98]. The predominant immunological alterations observed in the context of gut dysbiosis pertain to the functional reconfiguration of dendritic cells and glial cells within the CNS, in addition to the host's immune cells, such as effector T and B lymphocytes residing in the intestines, peripheral blood, and PNS [99]. Moreover, the gut microbiome and its metabolic byproducts interact with diverse cellular constituents of the CNS by stimulating immune signaling cascades, including the inflammasome, type 1 interferon, MyD88-dependent, and NF-κB pathways [100]. Consequently, the persistent neuroinflammation instigated by the excessive accumulation of lymphocytes, cytokines, and chemokines results in the aggregation and deposition of misfolded proteins in proximity to neurons (Fig. 4) [101].

In innate immunity, the accumulation of misfolded proteins and amyloid aggregates resembles PAMPs and thymus-independent type 2 (TI-2) antigens. PAMPs represent highly conserved molecular entities originating from microbial sources, including LPS, proteins, lipoteichoic acids, nucleic acids, double-stranded RNAs (dsRNAs), and CpG motifs, which are identified as significant targets of pathogenic importance by pattern recognition receptors (PRRs) within the innate immune system of the host [102]. The gut microbiota, along with their metabolic byproducts, engage in continuous interactions with PRRs expressed on peripheral blood immune cells, intestinal epithelial cells (IECs), blood endothelial cells (ECs), as well as neurons and glial cells (microglia and astrocytes) located within both the CNS and PNS [103]. TI-2 antigens exhibit comparable polymeric structures that directly induce the secretion of immunoglobulin M (IgM) by B-lymphocytes. Numerous experimental investigations have elucidated that amyloid-like deposits or protein aggregates may operate similarly to PAMPs, thereby inciting chronic activation of the innate immune response through their interaction with various PRRs, including TLRs, formyl peptide receptors, receptors for advanced glycation end products, scavenger receptors, components of the complement cascade, and pentraxins [104, 105]. In a milieu characterized by chronic oxidative stress, elevated concentrations of reactive oxygen and nitrogen species stimulate the release of pro-inflammatory mediators, such as cytokines and chemokines, neoepitopes, and damage-associated molecular patterns (DAMPs), which subsequently activate microglia and astrocytes [97, 106]. Furthermore, the dysregulated nature of the innate immune response renders microglia, exhibiting an immature phenotype and altered gene expression, incapable of efficiently clearing misfolded protein aggregates, a deficiency attributable to compromised autophagic mechanisms; this perpetuates a detrimental cycle characterized by the secretion of pro-inflammatory cytokines associated with a toxic cascade and resultant neuronal death [107].

The adaptive immune response is crucial for the defense against microorganisms and for maintaining equilibrium within the gut microbiota and host metabolism, thereby preventing an exaggerated immune response to innocuous substances. Dysregulation within the gut can adversely influence NDDs by generating neuroactive metabolites such as SCFAs and neurotransmitters and stimulating inflammatory mediators, including cytokines and gut peptides [108, 109]. Under pathological conditions, these metabolites may traverse the compromised BBB and subsequently instigate or amplify inflammatory processes within the brain by recruiting peripheral myeloid cells [110]. This, in turn, promotes the activation of microglia and astrocytes, resulting in a detrimental harmful cycle [111]. Furthermore, an imbalance of dysbiotic bacteria is correlated with an increased presence of reactive T lymphocytes in both the bloodstream and central nervous system, thereby obstructing the migration and proliferation of T cells in specific regions of the brain [112]. Such phenomena result in reduced CD4^+^ and CD8^+^ T cell subsets, impaired development of memory CD8^+^ T cells, decreased survival rates of Treg cells, and heightened Th2 and Th17 responses within the organism and CNS. These modifications facilitate the recruitment of dendritic cells, group 3 innate lymphoid cells, and granulocytes into the CNS [113]. Signals indicative of dysbiosis originating from the gastrointestinal tract incite an overproduction of IL-23 within Th17 cells while concurrently diminishing the immunosuppressive protective signals mediated by the expression of GATA binding protein 3 (GATA3), forkhead box protein P3 (Foxp3), and IL-33 within Treg cells, resulting in the chronic neuroinflammation characteristic of autoimmune disorders and NDDs [114, 115]. Moreover, the dysbiosis of the gut is known to activate the secondary immune response via B cells, thereby influencing the class switching of IgA in B cells [116]. Consequently, the microbiome and its metabolic byproducts exert a significant and reciprocal influence on the host's immune system, while gut dysbiosis predominantly alters the host's immune response through the activation of inflammasome signaling.

The adaptive immune response, encompassing both T- and B-lymphocytes, is posited to play an early etiological role in CNS autoimmune pathologies, including ALS and MS. In the context of AD and PD, the activation of innate immune responses, typically considered protective, serves to perpetuate pro-inflammatory stimuli [117]. Alterations in the functional dynamics of microglia and astrocytes further exacerbate neuronal loss and dysfunction, ultimately culminating in neurodegenerative processes [117, 118]. Treg cells are hypothesized to assume critical responsibilities within neurodegenerative diseases such as AD, PD, and ALS (Fig. 4). Recent investigations into CNS-related pathologies indicate that gut dysbiosis may represent a principal initiating factor for immune-mediated chronic inflammation, facilitating the translocation of misfolded proteins from the PNS to the CNS through the GBA network [99, 119]. These cascading events elicit alterations in immune activation and impede protein clearance, thereby engendering a neurotoxic inflammatory milieu that exacerbates the dissemination of protein aggregates across diverse cerebral regions, engendering a self-perpetuating cycle of immune-mediated inflammation and neuronal demise.

Numerous scholarly investigations have elucidated a correlation between neurodegeneration and the dysregulation of the GBA stemming from gut dysbiosis [120]. Gut dysbiosis associated with the majority of NDDs results in compromised autophagy across various cellular types (e.g., dendritic cell (DCs), IECs, T cells, ECs, B cells, glial cells, and neuronal cells), impaired protein clearance, misfolding of proteins, and the precipitating heightened oxidative stress, which subsequently generate either small oligomeric or large fibrillary aggregates. These misfolded aggregates activate PRRs, leading to inflammatory responses within the gut and CNS. Furthermore, the proliferation of gut pathobionts, including *Escherichia coli*, *Mycobacterium tuberculosis*, and *Klebsiella pneumonia*, contributes to increased production of amyloid proteins, which may act as potential nucleating agents in the misfolding of amyloid-beta (Aβ) within AD models [121]. Alterations in SCFA profiles attributable to gut dysbiosis, diminished mucin synthesis, and enhanced bacterial or antigen translocation further propagate systemic inflammation and the activation of microglia or enteric glia, both of which have been implicated in the misfolding of αSyn in PD models [122, 123]. Overall, the dysregulation of T helper cells, Treg cells, and DCs within the intestinal milieu, alongside dysfunctional microglia and Treg cells in the cerebral context, instigates a deleterious inflammatory cascade that ultimately results in neuronal cell death [124, 125]. These findings delineate the intricate interplay between gut dysbiosis-mediated amyloid-like deposition, impaired autophagy, altered immune homeostasis, and the pathophysiology of NDDs.

### Relationship between gut microbiota-immune interactions and NDDs

Bidirectional communication in the gut microbiota-brain axis is a sophisticated process that plays a critical role in maintaining the balance of gastrointestinal and brain functions. Numerous studies have demonstrated that the interaction between the immune system and the microbiome contributes to NDDs such as AD, PD, MS, ALS, and HD. This section provides a comprehensive review of these findings.

### Alzheimer’s disease (AD)

AD is a common form of senile dementia and cognitive impairment prevalent in older individuals. Currently, AD is on the rise due to changing demographic trends in modern societies. It is estimated that less than 2% of individuals under the age of 60 are affected by AD, while the prevalence rises to about 50% in those aged over 85. In 2010, approximately 36 million people globally were living with dementia, and projections indicate that this number will double every 20 years to reach 66 million by 2030 and 115 million by 2050 [126]. Several molecular mechanisms have been proposed to explain the neuropathological features of AD, linking neuronal toxicity to Aβ and tau proteins. These mechanisms include the disruption of Ca^2+^ homeostasis, oxidative stress, lipid metabolism, enzymatic deregulation, an impaired cell stress response, mitochondrial dysfunction, epigenetic changes, reduced cytoskeletal integrity, variations in Notch signaling, neuroinflammation, apoptosis, and the failure of multiple neurotransmitter pathways [1, 127].

The initial stages of AD are thought to be driven by the accumulation of Aβ proteins, while neuroinflammation influences the progression of cognitive decline [128]. Microglia expresses TLRs that detect soluble Aβ peptides and activate inflammasome complexes, initiating neuroinflammatory reactions [129]. Furthermore, type I interferon responses from peripheral immune cells, such as T cells, contribute to brain neuroinflammatory responses [130]. In fully developed neurons, the microtubule-associated tau protein is predominantly found within axonal structures, where it plays a crucial role in maintaining the integrity of the microtubule architecture and neuronal synapses. In the context of AD, improperly folded and hyperphosphorylated tau proteins accumulate within neurons, a phenomenon linked to impaired protein turnover at synaptic sites [131]. Current research indicates that Aβ proteins produced by particular gut microbiota, such as *Staphylococcus, Mycobacteria*, *Salmonella*, *Streptococcus*, *Klebsiella*, *Bacillus species*, and *Citrobacter*, may instigate the misfolding of proteins into Aβ conformations within the cerebral context, thereby exacerbating neuroinflammatory responses [121, 132]. A novel transgenic mouse model designated ADLP^APT^, has elucidated the occurrence of Aβ plaques, neurofibrillary tangles, and reactive gliosis in the cerebral tissue, concomitant with cognitive impairments, diminished intestinal barrier functionality, intestinal inflammation, and alterations in gut microbiota composition. Notably, these pathological changes were significantly mitigated by fecal microbiota transplantation (FMT) derived from healthy WT mice [133]. Similarly, following the administration of broad-spectrum antibiotics, cerebral analyses of APP/PS1 transgenic mice—an experimental model that simulates AD with concomitant gut dysbiosis (APPswe/PS∆E9)) (PAP)—revealed a pronounced decline in both the composition and diversity of the gut microbiota as well as diminished SCFA levels. Moreover, there were significant reductions in the concentrations of CD11b, COX-2, Aβ peptides, tau proteins, glial reactivity, and circulating cytokines and chemokines when compared to the WT control cohort [134]. Administering *L. plantarum* prevented cognitive dysfunction and increased serotonin, dopamine, and GABA levels. These changes ameliorated pathological processes by simultaneously modulating the gut microbial communities to alleviate gut dysbiosis and alleviate neuronal damage. Furthermore, these changes inhibited Aβ deposition and tau hyperphosphorylation by regulating the PI3K/Akt/GSK-3β pathway [135]. Likewise, diminished microglial activation within APP/PS1 mice facilitated increased Aβ deposition, synaptic impairment, and neuroinflammatory processes, resulting in cognitive deficits relative to WT mice. Nevertheless, transgenic mice that underwent FMT from WT counterparts demonstrated reductions in levels of Aβ and tau proteins, enhancements in synaptic plasticity, and modifications in gut microbiota composition compared to WT mice [136].

Clinical research has shown that individuals with AD have an altered gut microbiota and reduced microbial diversity, with decreased levels of beneficial gut bacteria such as *Bifidobacterium*, *Eubacterium rectale*, and *Dialister*. Conversely, there is an increase in harmful gut bacteria, including *Bacteroides*, *Escherichia/Shigella*, and *Ruminococcus* [137, 138]. In a recent study, researchers found that AD patients had lower *Bifidobacterium breve* strain A1 levels and higher *Escherichia/Shigella* bacteria concentrations. This imbalance was linked to higher levels of pro-inflammatory cytokines (IL-1 and CXCL2), which could lead to inflammatory responses in both the bloodstream and the brain [137, 138]. A recent clinical investigation revealed a notable increase in Aβ precursor protein, CD68, Aβ load, and β-Tau immunoreactivity in the intestines of AD patients and APP/PS1 mice. This suggests that the gut environment may mirror conditions in the brain, contributing to inflammation and dysregulated immune activation associated with Aβ precursor protein and Aβ pathology [139]. Reports indicate that AD patients have elevated lipopolysaccharide (LPS) levels and the *E. coli* K99 pili protein in their cerebral tissue and vascular systems. Additionally, the co-localization of LPS with Aβ1-40 within amyloid plaques suggests that bacterial byproducts from the gut may move to the brain through systemic circulation [140].

### Parkinson’s disease (PD)

PD is the second most common movement disorder caused by neurodegeneration, and it is known for its diverse motor symptoms such as muscle stiffness, tremors, difficulty walking, and slow movement, and non-motor symptoms including depression, cognitive decline, gastrointestinal issues, sleep disturbances, loss of smell, cardiovascular problems, constipation, and urogenital abnormalities. Recent research indicates that PD affects over 10 million individuals globally [141]. The main pathological features of PD involve the accumulation of the α-synuclein protein and the loss of neuronal cells, particularly dopamine-secreting neurons in the brain. The connection between PD and autoimmune disorders has been proven through compromised cellular and humoral immune reactions and immune system imbalances [142]. Furthermore, research has consistently shown that people with PD often suffer from gastrointestinal issues, including neurodegeneration, increased intestinal permeability, inflammation, and disruptions in the balance of the gut microbial flora. These factors are thought to contribute to the development and progression of the disease [143]. Most PD patients experience worsened intestinal permeability, which could indicate imbalances in the gut microbiota. Intestinal permeability has been linked to the exacerbation of motor impairment, microglia activation, and the development of α-synuclein pathology [144, 145].

A recent study found that administering FMT to mice with PD restored the gut microbiota, improving gastrointestinal function and motor skills and reducing intestinal inflammation and damage to the gut barrier, thus resulting in lower levels of systemic inflammation. Subsequently, FMT treatment alleviated BBB dysfunction and diminished neuroinflammation within the substantia nigra (SN), which consequently led to a reduction in the degeneration of dopaminergic neurons. Further investigation demonstrated that FMT treatment decreased LPS concentrations in the colon, serum, and SN, impeding the TLR4/MyD88/nuclear factor kappa B (NF-κB) signaling cascade and its corresponding pro-inflammatory mediators in the SN and colon [146]. A recent study employing a mouse model of PD induced by 1-methyl-4-phenyl-1,2,3,6-tetrahydropyridine (MPTP) suggests that FMT derived from healthy donors can reverse modifications in gut microbiota composition. Furthermore, these PD-afflicted mice exhibited enhanced levels of SCFAs, striatal dopamine, and 5-HT, along with diminished physical disabilities. FMT also conferred protection to PD mice by attenuating the activation of microglia and astrocytes and the TLR4/tumor necrosis factor-alpha (TNF-α) signaling pathway in the gut and brain [147]. In another investigation, *L. plantarum* was found to decrease the accumulation of α-synuclein in the SN, promote the expression of the nuclear factor erythroid 2-related factor 2 (Nrf2)/antioxidant response element (ARE) and peroxisome proliferator-activated receptor gamma coactivator-1 (PGC-1α) pathways, and inhibit the pro-inflammatory cytokines and NOD-like receptor protein 3 (NLRP3) inflammasome. Furthermore, an analysis of fecal samples showed that *L. plantarum* altered the gut microbiota composition in PD mice by decreasing the population of harmful bacteria such as *Proteobacteria* and *Actinobacteria* and increasing the presence of beneficial bacteria like *Lactobacillus* and *Prevotella* [148].

In a PD mouse model study, the gut microbiota was shown to control pathways that contribute to the accumulation of α-synuclein and impede the removal of insoluble protein aggregates [145]. Therefore, potential treatments for PD have been developed, including immunotherapy targeting α-synuclein and immune mediators [149]. Patients with PD were observed to have an excessive abundance of bacteria growth in the small intestine, a phenomenon correlated with dysfunctional absorption and motor deficits. Mice possessing the gut microbiota from PD patients exhibited exacerbated motor dysfunction. In comparison to healthy individuals, PD patients were characterized by diminished levels of *Bacteroides*, *Prevotella*, *Butyricicoccus*, *Pepto-streptococcus*, and *Lactobacillus* spp., alongside enhanced levels of *Enterobacter*, *Lactobacillus*, and *Proteus* species [150]. A clinical analysis of fecal samples showed significant changes in the gut microbiota composition of PD patients compared to age-matched controls. In particular, *Bacteroidetes* and *Prevotellaceae* were less abundant in PD patients, who exhibited a greater abundance of *Enterobacteriaceae* and *Lactobacillaceae*. These changes correlate positively with increased postural instability and a distinctive gait [151, 152].

### Multiple sclerosis (MS)

MS is an inflammatory demyelinating disease linked to dysfunction in the immune system and gut microbiome. T cells play a crucial role in regulating the nervous system, and their immune activity can either exacerbate abnormal autoimmune reactions or lead to CNS inflammation, worsening MS symptoms. Recent investigations indicate that dysbiosis of the gut microbiota significantly contributes to the pathogenesis of MS. The relapsing/remitting variant of MS is typified by a decrease in beneficial bacterial populations alongside an elevation in pathogenic bacterial species, which adversely impacts the modulation of immune cell populations, including IL-10-producing CD4^+^ T cells, Treg cells, DCs, regulatory B cells, and suppressive macrophages [153].

EAE serves as an appropriate animal model for the study of MS and is characterized by an elevation in immune cell populations that secrete pro-inflammatory cytokines, particularly Th1 and Th17 cells. Recent research showed that gamma-aminobutyric acid (GABA), produced by *Lactobacillus brevis*, had inhibitory effects on both the growth and release of IFN-γ and IL-17 from mesenteric lymph node cells, as well as impacting the expression of costimulatory molecules on antigen-presenting cells (APCs). Conversely, GABA was discovered to enhance the expression of immunoregulatory markers, such as TGF-β, Foxp3^+^, and IL-10 [154]. Another recent investigation revealed that the antibiotic-induced depletion of the gut microbiota in a mouse model of EAE resulted in a reduction of motor impairments and axonal damage. Subsequent recolonization with the microbiota reinstated susceptibility to EAE by engaging T cells (CD4^+^ and CD39^+^) and B cells (CD1d^+^ and CD5^+^) [155]. Likewise, antibiotic treatment in EAE models postponed the emergence of clinical manifestations by diminishing serum levels of IFN-γ and IL-17A while augmenting IL-10 levels. Furthermore, antibiotic intervention resulted in reduced BDNF levels but increased TNF-α and IL-1β levels within the hippocampus of EAE-afflicted mice. Additionally, antibiotics were associated with a decrease in depression-like symptoms, an increase in anxiety-like behavior, and enhancement of cognitive function, including learning and memory [156]. Rats exhibiting greater diversity of *Lactobacillus* species demonstrated a decreased likelihood of developing EAE compared to those with a lower abundance of lactic acid bacteria. This observation suggests that the enhancement of gut microbiota through the incorporation of beneficial Lactobacillus strains could serve as a preventive measure against MS [157].

Prior research has indicated that raising serotonin levels may lead to immune-modulating effects and delay the advancement of MS/EAE. For instance, elevated serotonin levels could reduce disease severity by diminishing the proliferation of T cells, suppressing the secretion of IL-17 and IFN-γ, and promoting the production of IL-10 [158]. *Acinetobacter calcoaceticus* and *Akkermansia muciniphila*, both of which are found to be elevated in patients with MS, provoked pro-inflammatory responses in human peripheral blood mononuclear cells as well as in mice colonized with these bacterial species. Conversely, *Parabacteroides distasonis*, which has been observed to be diminished in MS patients, activated the differentiation of anti-inflammatory IL-10-expressing human IL-10^+^, FoxP3^+^, Tregs cells, and CD4^+^, CD25^+^, T cells in mouse models. Moreover, the transplantation of microbiota-derived from MS patients into GF mice resulted in more severe EAE symptoms and a lower frequency of IL-10^+^ Tregs than mice that were "humanized" with microbiota from healthy control subjects [159].

### Amyotrophic lateral sclerosis (ALS)

ALS is a progressive NDD that primarily affects upper and lower motor neurons. Typically, motor functions in ALS patients deteriorate within three to five years, often leading to respiratory failure in the advanced stages of the disease [160]. Approximately 35% of ALS patients experience concurrent cognitive impairments, which primarily present as visual-spatial or language disorders or impaired executive function. A subset of ALS patients may also exhibit behavioral impairments such as apathy, loss of interest, irritability, and aggression [160]. Research suggests that neuroinflammation may play a role in ALS progression, as evidenced in animal models and individuals with ALS. This inflammation contributes to disease progression and may be linked to disease onset. Activation of glial cells leading to neuroinflammation is associated with motor neuron loss, whereas lower levels of regulatory Tregs (which help suppress inflammation) are inversely correlated with the rate of ALS progression [161].

The gut microbiota is thought to impact ALS by regulating inflammation and energy metabolism pathways, suggesting that it could be involved in the disease’s development. ALS pathogenesis is associated with changes in the gut microbiota composition, impaired metabolism, altered innate immune responses, and the production of gut-derived neurotoxins (such as tetanus and botulinum toxins) by *Clostridium* spp., which are associated with ALS pathogenesis leading to brain damage [162]. SOD1^G93A^ transgenic mice, commonly used as an animal model of ALS, exhibit increased intestinal permeability and reduced levels of butyrate-producing bacteria. Butyrate is an anti-inflammatory SCFA that results from bacterial fermentation of indigestible fiber in the colon [163]. The findings of Blacher et al. [164] revealed that transgenic SOD1^G93A^ mice exhibited changes in their microbial composition and metabolite configuration prior to the onset of motor symptoms. The disease severity worsened when the mice were treated with antibiotics under GF conditions. The presence of specific strains, including *A. muciniphila*, *Prevotella melaninogenica*, *P. distasonis*, *Lactobacillus gasseri*, and *Ruminococcus torques*, was associated with increased disease severity. Notably, *A. muciniphila* abundance decreased as the disease progressed. Conversely, supplementation with *A. muciniphila* improved ALS symptoms, while *R. torques* and *P. distasonis* exacerbated symptoms in transgenic mice. Additionally, providing 2% butyrate in drinking water to an ALS mouse model enhanced gut integrity and survival [163].

### Huntington’s disease (HD)

HD is a harmful genetic disorder that affects both men and women. HD leads to motor, cognitive, and psychiatric symptoms, as well as disruptions in gut function and unintended weight loss. The mutated huntingtin protein forms clusters in the brain, causing dysfunction and neuron death [165]. The cerebral cortex, hippocampus, and striatum represent the principal brain regions adversely impacted, leading to a depletion of distinct neuronal subtypes. The precise mechanisms underlying neurodegeneration in HD remain inadequately elucidated; however, numerous pathways have been identified as potential contributors, including transcriptional dysregulation, oxidative stress, mitochondrial dysfunction, compromised protein clearance, and excitotoxicity [166].

While the majority of investigations focus on the brain and neuropsychiatric manifestations, peripheral elements such as the gut are also believed to play a vital role in the pathology of the disease. Evidence indicates that the gut microbiota and its interplay with the brain should be considered a significant contributing factor in the trajectory of disease progression. Moreover, dysbiosis of the gut microbiota may lead to an increase in the pro-inflammatory microbial metabolite’s synthesis alongside a diminished synthesis of anti-inflammatory metabolites, such as SCFAs, thereby exacerbating neuroinflammation and immune dysregulation [167].

In a recent study using the R6/1 transgenic mouse model of HD, researchers found that the gut microbiota composition changed at 12 weeks of age: the number of Bacteroidetes increased while that of Firmicutes decreased. This imbalance in gut bacteria was associated with slower growth despite consuming more food and motor deficits [168]. Another R6/2 mouse model of HD demonstrated improved intestinal permeability and gut dysbiosis and a notable reduction in colon length compared to normal WT littermates. In addition, the gut microbiota of R6/2 mice exhibited an increased abundance of *Bacteroidetes* and a diminished relative abundance of *Firmicutes*. At 18 weeks, the colon mucosa of R6/2 mice displayed decreased occludin expression, resulting in disrupted epithelial structure. In contrast, WT mice demonstrated robust expression of mucosal occludin alongside preserved epithelial integrity [169]. Patients with HD exhibited marked differences in their gut microbial communities (beta diversity) relative to healthy controls and diminished species richness and evenness (alpha diversity) [167].

Du et al. [170] reported that patients with HD exhibited elevated α- and β-diversity of gut microbiota compared to healthy controls. They noted that the abundance of the genus *Intestinimonas* was positively correlated with plasma levels of IL-4. In contrast, the abundance of the genus *Bilophila* exhibited a negative correlation with pro-inflammatory IL-6 levels. These observations imply that alterations in the fecal microbiota may influence the immune responses of individuals affected by HD. Furthermore, after antibiotic pretreatment, FMT from healthy WT mice to HD R6/1 mice substantially enhanced cognitive functions but only in female mice. This enhancement may be attributable to acetate imbalance, microbial instability, and divergent gut immune profiles observed in male subjects [171].

## Potential therapeutic approaches targeting the microbiota to treat NDDs

### Probiotics and prebiotics

*Bifidobacterium* and *Lactobacillus* represent the most predominant probiotic microorganisms identified in dairy products such as yogurt and various pharmaceutical formulations. A research investigation conducted by Tamtaji et al. [172] highlighted that the simultaneous administration of probiotics (specifically *B. bifidum*, *L. acidophilus*, and *B. longum* at a dosage of 2 × 10^9 CFU/day) in combination with selenium supplementation markedly improved cognitive performance in patients with AD when compared to the effects observed with selenium or placebo treatment alone. The introduction of the probiotic strains *Bifidobacterium* and *Lactobacillus* further alleviated constipation and motor dysfunction while demonstrating significant systemic impacts on inflammation and metabolic processes in patients diagnosed with PD [173]. An experimental study utilizing an animal model indicated that a 4-week regimen involving *Clostridium butyricum* (at a dosage of 5 × 10^8 CFU/day) resulted in a reduction of cognitive deficits and inhibited the accumulation of Aβ deposits, activation of microglia, as well as the synthesis of pro-inflammatory cytokines such as TNF-α and IL-1β; furthermore, it diminished microglial-mediated neuroinflammatory responses in the cerebral regions of APP/PS1 transgenic mice. Additionally, treatment with *C. butyricum* reinstated the dysregulated gut microbiome and restored butyrate concentrations. Importantly, butyrate administration decreased the expression of COX-2 and CD11b, along with the attenuation of NF-κB p65 phosphorylation in Aβ-stimulated BV-2 microglial cells [174]. Similarly, the administration of *C. butyricum* also ameliorated motor dysfunctions, neurodegeneration of dopaminergic neurons, synaptic impairments, and microglial activation in murine models subjected to MPTP-induced PD. These therapeutic outcomes were associated with reestablishing gut microbiota and reducing colonic G-protein-coupled receptors 41/42, colonic glucagon-like peptide-1 (GLP-1), and cerebral GLP-1 receptors [175].

**Figure 5.**
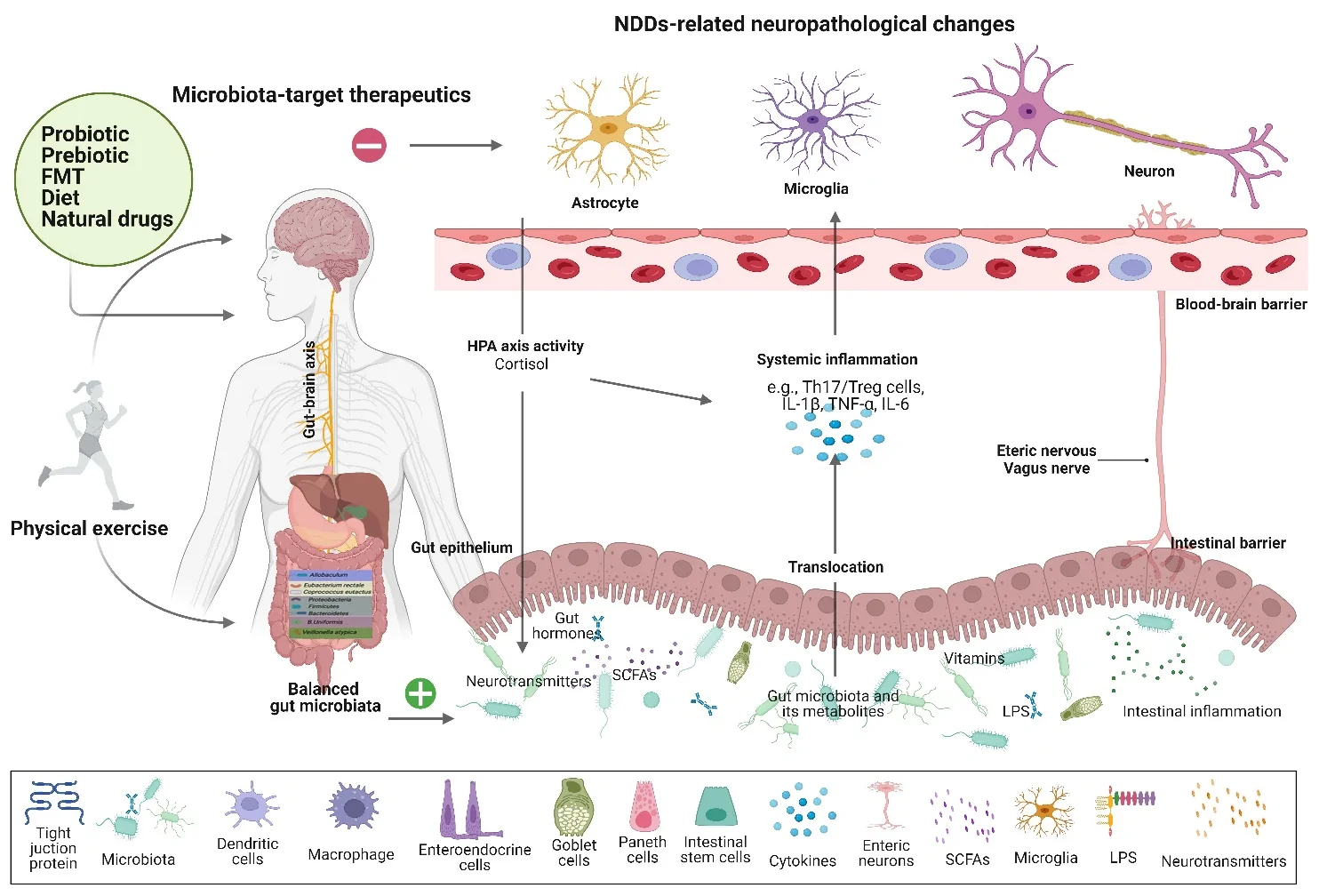
**The microbiota-gut-brain axis has the potential to play a role in the development of NDDs and microbiota-targeting therapies for these conditions**. This axis involves neural circuitry (such as the ENS, vagus nerve, neurotransmitters, and neuroactive metabolites like SCFA), neuroendocrine pathways (including the release of gut hormones like neuropeptides and cortisol through the HPA axis), and immune system pathways (such as cytokines). Neuroactive compounds in the diet and metabolites produced by the microbiota have regulatory effects on the microbiome-gut-brain axis, impacting hormone secretion from enteroendocrine cells, gut barrier function, neurotransmitter production by gut epithelial cells and microbiota, as well as signaling by enteric glial cells. All of these factors are relevant to the underlying mechanisms of NDDs. The (+) symbol indicates that neuroactive compounds, which include probiotics, prebiotics, dietary supplements, natural molecules, and metabolites produced by microbes, along with physical activity, can regulate or stimulate the microbial composition, promote the microbiome-gut-brain axis communication, and maintain gut-brain health. The (-) symbol indicates reduced severity of NDD-related neuropathological changes. It was created with BioRender.com. SCFAs, Short-chain fatty acids; HPA, Hypothalamic-pituitary-adrenal; IL-6, Interleukin-6; TNF-α, Tumor necrosis factor-alpha; IL-1β, Interleukin-1β; LPS, Lipopolysaccharide; FMT; Fecal microbiota transplantation.

Over two months, the intake of soluble fiber significantly attenuated astrocyte activation and enhanced cognitive function in six-month-old male AD APP/PS1 mice. These beneficial effects were closely linked to butyrate and propionate production by the gut microbiota. However, such effects were not evident in mice subjected to antibiotic treatment, suggesting that the neuroprotective efficacy of soluble fiber is contingent upon a healthy gut microbiome [176]. A 16-week dietary regimen rich in fiber led to improvements in motor capabilities and a reduction in α-synuclein aggregation within the substantia nigra of transgenic mice exhibiting PD, which was achieved through the reversal of detrimental microglial activation states (Fig. 5) [177]. The neuroprotective potential of various probiotic strains (e.g., *Bifidobacterium* and *Lactobacillus* spp.) has been explored in animal models of HD [172]. Effective microorganisms include *L. rhamnosus*, *Bifidobacterium*, *L. casei*, *L. reutri*, *L. acidophilus*, and *Saccharomyces*. Gut dysbiosis is always linked to a worsened immune system, which can lead to various infections. Research has shown that probiotic intake increases the production of natural killer cells and other immune response-related cells. Probiotics help regulate inflammatory responses and enhance the epithelial barrier’s function. They also have the potential to boost SCFA production. Hence, probiotics contribute to maintaining the health of the host organism [178].

Recent studies on AD suggest that probiotic supplementation could help alleviate HD-related symptoms by modulating neurotransmitter production, reducing neuroinflammation, and promoting neurogenesis [179]. For instance, a high-fiber diet enhanced cognitive and emotional functions and improved gastrointestinal function in mice with HD by changing the gut microbiota composition [180]. Another study reported that administering *L. acidophilus*, *B. bifidum*, *L. casei*, and *L. fermentum* to patients with MS improved their mental health, insulin resistance, and high-density lipoprotein (HDL) and total/HDL cholesterol levels and downregulated gene expression of IL-8 and TNF-α mRNA in peripheral blood mononuclear cells [181]. According to Asghari et al. [182], administering a probiotic supplement composed of *Saccharomyces boulardii* for four months significantly enhanced oxidative stress indicators, inflammatory markers, fatigue levels, pain perception, and overall quality of life among patients diagnosed with MS. The administration of probiotics, including strains of *Lactobacillus*, Streptococcus, and *Bifidobacterium*, twice daily for two months elevated an anti-inflammatory response within the peripheral immune system. It resulted in a reduction of human leukocyte antigen D-related dendritic cells. Furthermore, this intervention correlated with an increased abundance of *Bifidobacterium* and *Lactobacillus*, which is associated with a mitigated risk of MS (Fig. 5) [183]. Studies on the neuroprotective effects of probiotics and prebiotics in gut-microbiota modulation are presented in Table 1.

**Table 1:** Potential therapeutic approaches targeting probiotics and prebiotics to treat NDDs.

Microecological agents	Impact on microbiota	Therapeutic effects	References
*B. bifidum*, *L. acidophilus*, and *B. longum* in combination with selenium	Systemic impacts on inflammation and metabolic processes	Improved cognitive performance and alleviated constipation and motor dysfunction	[173]
*C. butyricum*	Restored gut microbiome and butyrate concentrations	Improved cognitive function, inhibited Aβ accumulation, and reduction of microglia-mediated neuroinflammation	[174]
*C. butyricum*	Reestablishing gut microbiota and reducing colonic G-protein-coupled receptors 41/42, GLP-1, and cerebral GLP-1 receptors	Ameliorated motor dysfunctions, neuronal loss, synaptic impairments, and microglial activation	[175]
Soluble fiber	Promote the butyrate and propionate production by the gut microbiota	Attenuated astrocyte activation and enhanced cognitive function	[176]
Soluble fiber	Improved healthy gut microbiome	Improve the motor capabilities and a reduction in α-synuclein aggregation and reversal of microglial activation	[177]
*L. rhamnosus*, *Bifidobacterium*, *L. casei*, *L. reutri*, *L. acidophilus*, and *Saccharomyces*	Enhance the epithelial barrier’s function, boost SCFAs production, and maintain the host organism	Increases the production of natural killer cells and regulates inflammatory responses	[178]
*L. plantarum*	Modulating neurotransmitter production	Alleviate HD-related symptoms, reducing neuroinflammation and promoting neurogenesis	[179]
High soluble fiber	Improved gastrointestinal function and changing the gut microbiota composition	Enhanced cognitive and emotional functions	[180]
*L. acidophilus*, *B. bifidum*, *L. casei*, and *L. fermentum*	Downregulated gene expression of IL-8 and TNF-α mRNA in peripheral blood mononuclear cells	Improved mental health, insulin resistance, and HDL and total/HDL cholesterol levels	[181]
*S. boulardii*	Maintain the host organism's	Enhanced oxidative stress indicators, inflammatory markers, fatigue levels, and pain perception	[182]
*Lactobacillus*, Streptococcus, and *Bifidobacterium*	Reduction of human leukocyte antigen D-related dendritic cells	Elevated anti-inflammatory response and mitigated risk of MS	[183]

Aβ: amyloid beta, GLP-1: glucagon-like peptide-1, HD: Huntington's disease, HDL: high-density lipoprotein, IL-8: interleukin-8, MS: Multiple sclerosis, mRNA: messenger RNA, SCFA: short-chain fatty acids, TNF-α: Tumor necrosis factor-alpha.

### Fecal microbiota transplantation (FMT)

FMT represents a promising strategy for restoring the gut microbiota. The transfer of diverse microbial species and their metabolic products from healthy donors may augment microbial diversity, modulate metabolite synthesis by specific microbial taxa and the host organism, and influence the host’s immune response. Investigations employing murine models have demonstrated that FMT from healthy WT mice, following pretreatment with an antibiotic regimen, can ameliorate abnormalities in the colonic expression of genes associated with gut macrophage functionality and circulating inflammatory monocytes. Therefore, the cerebral accumulation of Aβ and the phosphorylation of tau protein are reduced, enhancing cognitive performance in AD models [136]. An additional study indicated that FMT from either young or healthy WT mice, administered without prior antibiotic intervention, alleviated Aβ plaque deposition and cognitive deficits in the 5×FAD mouse model [184]. Furthermore, notable improvements were observed two weeks after the administration of FMT from healthy donors devoid of prior antibiotic treatment in rodent models exhibiting motor deficiencies induced by rotenone. These beneficial outcomes may be attributable to the attenuation of LPS and the TLR4/myeloid differentiation primary response gene 88/NF-κB signaling cascade in the colon, serum, and CNS, alongside enhanced BBB integrity (Fig. 5) [146].

Nevertheless, few clinical cases or trials evaluating the impact of FMT on patients with AD and PD have yielded encouraging findings. In one study, dementia patients experiencing cognitive decline concomitant with *Clostridioides difficile* infection underwent FMT via colonoscopy 48 hours after the cessation of antibiotic therapy (with vancomycin and metronidazole). These patients exhibited improvements in cognitive function relative to baseline measurements one-month post-FMT from healthy donors [185]. Recently, Cheng et al. conducted a study involving 54 PD patients, of whom 27 were administered oral FMT capsules, and 27 received placebo capsules devoid of antibiotic pretreatment. The cohort undergoing oral FMT demonstrated significant improvements in PD-related autonomic symptoms, gastrointestinal disorders, and the complexity of the microecological system three months post-intervention [186]. In another investigation of 15 PD patients, 10 received colonic FMT, while five were administered nasointestinal FMT. The FMT intervention remedied motor deficits, quality of life, and sleep and alleviated anxiety and depression one and three months after the procedure. Nonetheless, five of the 15 patients reported adverse effects, including diarrhea, flatulence, and abdominal pain. Furthermore, colonic FMT demonstrated superior efficacy compared to nasointestinal FMT and sustained its therapeutic benefits for an extended duration [187].

Several case studies have highlighted the potential benefits of FMT for patients with MS. Three patients experienced significant improvements, such as regaining the ability to walk, resolution of long-standing symptoms, and becoming symptom-free after FMT. Subsequent studies since 2018 have supported these findings, showing either stabilization or improvement in disability scores, relief from gastrointestinal issues, and enhanced motor function. Moreover, an increase in butyrate-producing bacteria and SCFAs was observed after FMT, suggesting a potential therapeutic mechanism [188-190]. FMT rectified gastrointestinal dysbiosis in the HD mouse model and ameliorated cognitive impairments [171]. Moreover, a 48-year-old female who was diagnosed with ALS saw an improvement in symptoms after experiencing washed microbiota transplantation (WMT), a modified form of FMT. This not only helped to stabilize their ALS symptoms but also indicated the potential of WMT as a promising therapy for ALS (Fig. 5) [191]. Studies on the neuroprotective effects of FMT in gut-microbiota modulation are presented in Table 2.

### Diet

Extensive research has revealed a significant correlation between dietary habits and the risk factors associated with NDDs such as AD, PD, HD, MS, and ALS. Diet plays a significant role in modulating the composition of microbial communities within the gastrointestinal tract, and alterations in dietary habits can directly influence both the structure and functionality of gut microbiota by affecting the availability of macro- and micronutrients. Comprehensive preclinical and clinical investigations have been undertaken to explore the intricate relationship between dietary regulation of the gut-brain axis and the compositional shifts within the gut microbial ecosystems [192].

A recent investigation revealed that mice subjected to a KD over a period of 16 weeks exhibited enhanced cerebral blood circulation along with an increased relative abundance of beneficial microorganisms, including *Akkermansia muciniphila* and *Lactobacillus*. Moreover, there was a notable reduction in the levels of potentially pro-inflammatory bacterial species such as *Desulfovibrio* and *Turicibacter*, which subsequently inhibited the mammalian target of rapamycin (mTOR) signaling pathway through the activation of endothelial nitric oxide synthase (eNOS), resulting in improved neuro-vascularization [193]. Furthermore, the secretion of (D)-3-hydroxybutyrate ketone bodies, which is influenced by dietary interventions, is modulated by the gut microbiota and possesses the capability to communicate with peripheral tissues and the brain via GPCR signaling and epigenetically regulated gene expression, thereby providing a protective effect against oxidative stress [193]. The four-month KD intervention led to diminished levels of pro-inflammatory cytokines (IL-12p70 and IL-1β), rectifying dysbiosis within gut microbial communities and affecting the BDNF signaling pathway, which is critically associated with cognitive function and brain function (Fig. 5) [194].

**Table 2:** Potential therapeutic approaches targeting the FMT to treat NDDs.

FMT	Impact on microbiota	Therapeutic effects	References
FMT	Ameliorate abnormalities in the colonic expression, gut macrophage functionality, and circulating inflammatory monocytes	Enhancing cognitive performance and reduced accumulation of Aβ and the phosphorylation of tau	[136]
FMT	-	Alleviated Aβ plaque deposition and cognitive deficits	[184]
FMT	Enhanced BBB integrity	Reduced motor deficiencies and attenuation of primary response gene 88/NF-κB signaling	[146]
FMT	-	Improve cognitive function	[185]
FMT	Reduced gastrointestinal dysfunctions	Improvements in PD-related autonomic symptoms	[186]
FMT	Maintain the host organism's and superior colonic efficacy	Alleviate motor deficits and reduce depression and anxiety	[187]
FMT	Increase in butyrate-producing bacteria and SCFAs	Enhanced motor function	[188-190]
FMT	Reduced gastrointestinal dysbiosis	Ameliorated cognitive impairments	[171]
FMT	-	Modulates ALS symptoms	[191]

Aβ: amyloid beta, ALS: Amyotrophic lateral sclerosis, BBB: blood-brain barrier, FMT: fecal microbiota transplant, SCFA: short-chain fatty acids, PD: Parkinsons disease.

Polyphenols, which are phytochemicals predominantly metabolized by the intestinal microbiota within the colon, exhibit potential benefits in the context of age-related pathologies, including NDDs [195]. Consequently, the European Association for the Study of Obesity and the European Association for the Study of NDDs have recommended a higher intake of polyphenols among individuals afflicted with disorders related to the gut-brain axis. For example, dietary polyphenols such as isoflavones and lignans, along with their microbial metabolites, can traverse the intestinal barrier and the BBB, thereby mitigating neuroinflammatory responses [196]. Additionally, the tea polyphenol (-)-epigallocatechin-3-gallate has been shown to attenuate the hypothalamic-pituitary-adrenal (HPA)-axis, augment the concentration of SCFAs, regulate gut-brain communication, and ameliorate age-related cognitive declines [197]. While certain nutritional interventions have demonstrated neuroprotective properties, further research is warranted to delineate the specific mechanisms through which these dietary strategies exert influence on the gut-brain axis (Fig. 5). Studies on the neuroprotective effects of dietary intervention in gut-microbiota modulation are presented in Table 3.

### Natural drugs

There exists a complex interplay between pharmacological agents and gut microbiota, with the gut-brain axis representing a potential pathway for the transmission of neuroprotective effects of these agents in relation to brain health. Anthocyanins derived from plant sources may mitigate behaviors associated with cognitive deficits through the rectification of dysbiosis, the restoration of the intestinal ecological and functional microenvironment, and the suppression of inflammation induced by immune cells [198]. In addition to ameliorating cognitive decline, oxymatrine derived from plant roots may alleviate both clinical symptoms and pathological features of MS by reducing the concentrations of isovaleric acid and isobutyric acid in EAE mice, which correlate significantly with the abundance of particular gut microbial species and align with a reduction in the expression levels of TNF-α, IL-6, and IL-1β, as well as diminished permeability of the BBB, while concurrently enhancing the expression of ZO-1 and occludin in the brains and colons of EAE mice [199]. Another natural phytochemical, pterostilbene, has demonstrated the capacity to mitigate cognitive dysfunction, improve neuronal integrity, and inhibit the accumulation of Aβ in mice exhibiting cognitive impairment secondary to diabetes. Moreover, the administration of pterostilbene has been shown to inhibit the activation of microglia and astrocytes by diminishing oxidative stress, resuting the release of pro-inflammatory cytokines, and modulating TLR4/NF-κB signaling pathways within both the colon and the brain. Additional investigations revealed that pterostilbene could also enhance the homeostasis of intestinal microbiota, elevate the concentrations of SCFAs and their receptors, prevent the loss of intestinal tight junctions, and modulate various metabolites and metabolic pathways associated with inflammation, thereby mitigating systemic inflammation in mice exhibiting cognitive impairment due to diabetes [200]. Du et al. [201] revealed that Lingguizhugan decoction, an ancient herbal formulation from Chinese medicine, attenuated cognitive decline, reduced Aβ deposition, and diminished the production of pro-inflammatory cytokines within the brain.

**Table 3:** Potential therapeutic approaches are targeting the dietary intervention to treat NDDs.

Dietary intervention	Impact on microbiota	Therapeutic effects	References
KD	Enhanced cerebral blood circulation and increased beneficial microorganisms	Reduction of oxidative stress and pro-inflammatory cytokines and inhibited mTOR signaling	[193]
KD	Reduced gastrointestinal dysbiosis and increased BDNF expression	Improves cognitive and brain function and reduces levels of IL-12p70 and IL-1β	[194]
Isoflavones and lignans	Intestinal barrier and the BBB	Mitigating neuroinflammatory responses	[196]
(-)-epigallocatechin-3-gallate	Attenuate the HPA axis and synthesis of SCFAs production	Ameliorate age-related cognitive declines	[197]

BBB: blood-brain barrier, BDNF: brain-derived neurotrophic factor, HPA: hypothalamic-pituitary-adrenal, IL-1β: interleukin-1 beta, IL-12p70: interleukin (IL)-12 family members, beta SCFA: short-chain fatty acids, mTOR: mammalian target of rapamycin.

Furthermore, Lingguizhugan boosted the integrity of both intestinal and brain tissue barriers, modified the intestinal microecology, and regulated the transport of Aβ through SCFAs produced by gut microbiota. It additionally inhibited phosphorylated P38 of the mitogen-activated protein kinase (MAPK) signaling pathway in an AD-like mice model. The microbiota-gut-brain axis serves as a nexus for multiple biofeedback mechanisms through which numerous pharmacological agents exert their effects on CNS disorders. For instance, a recent investigation indicated that dimethyl itaconic acid diminished synaptic plasticity damage, elevated the expression of BDNF, synapsin (SYN), and postsynaptic density protein 95 (PSD95) proteins, and inhibited neuroinflammation mediated by microglial activation in mice subjected to a high-fat diet (HFD). In the colonic context, supplementation with dimethyl itaconic acid significantly lowered the expression levels of pro-inflammatory cytokines (TNF-α, IL-1β, IL-6) while concurrently reducing macrophage infiltration in HFD-fed mice, as well as upregulating the expression of cytokines associated with immune homeostasis (IL-22, IL-23) and the antimicrobial peptide Reg3γ. Furthermore, dimethyl itaconic acid ameliorated the HFD-induced expression of tight junction proteins, reduced impairments to the gut barrier, and promoted the proliferation of propionate- and butyrate-producing bacteria through the improvement of microbiome composition [202]. Consequently, naturally occurring plant-derived bioactive compounds hold significant promise in the therapeutic management of NDDs through the modulation of gut microbiota and their associated microbial composition (Fig. 5). Studies on the neuroprotective effects of natural drug intervention in gut-microbiota modulation are presented in Table 4.

### Exercise

A growing body of evidence shows that physical activity alters the composition of the gut microbiota and associated metabolites, with such alterations providing health benefits and potentially hindering disease development [203]. Recent investigations over the past five years have demonstrated that physical activity promotes microbial diversity within the gut [204]. Furthermore, physical activity has been shown to diminish the duration of transient stool passage within the gastrointestinal tract, minimizing the interaction between pathogenic microorganisms and the mucosal layer of the gastrointestinal system; in turn, this reduces pathogen dissemination into the circulatory system and mitigates the effects of such populations. Recently, empirical evidence has identified a causal relationship between exercise and modulation of the gut microbiota, demonstrating health benefits through the colonization of GF murine models with microbiota derived from physically active mice in contrast to those colonized by sedentary counterparts, leading to enhancements in gut morphology, inflammatory response profiles, and the reaction to experimentally induced colitis [205]. Moreover, alterations in the gut microbiota composition may undermine the physiological functions of the CNS. As previously noted, physical activity has well-established brain health benefits. Furthermore, several studies have suggested that exercise may positively modify the intestinal flora diversity, improving the functionality of the gut-brain axis [203].

**Table 4:** Potential therapeutic approaches are targeting the natural drugs to treat NDDs.

Natural drugs	Impact on microbiota	Therapeutic effects	References
Oxymatrine	Reducing the concentrations of isovaleric acid and isobutyric acid and diminished permeability of the BBB	Alleviate both clinical symptoms and pathological features of MS by reduction of TNF-α, IL-6, and IL-1β	[199]
Pterostilbene	Enhanced intestinal microbiota homeostasis, increased SCFAs and their receptors, and prevented tight junction loss	Mitigate cognitive dysfunction, improve neuronal integrity, and inhibit the accumulation of Aβ by diminishing microglia and astrocyte activation and oxidative stress	[200]
Lingguizhugan decoction	Boosted the integrity of intestinal tissue barriers and synthesis of SCFAs concentrations	Attenuated cognitive decline, reduced Aβ deposition, and diminished the production of pro-inflammatory cytokines by inhibiting phosphorylated P38 of the MAPK signaling pathway	[201]
Dimethyl itaconic acid	Improve the gut barrier and promote propionate- and butyrate-producing bacteria	Improve synaptic plasticity, elevated expression of BDNF, SYN, and PSD95 proteins, and lowered TNF-α, IL-1β, and IL-6 expression	[202]

Aβ: amyloid beta, BBB: blood-brain barrier, BDNF: brain-derived neurotrophic factor, IL-6: interleukin-6, IL-1β: interleukin-1 beta, MAPK: mitogen-activated protein kinase, MS: Multiple sclerosis, PSD95: postsynaptic density protein 95, SCFA: short-chain fatty acids, SYN: synapsin, TNF-α: Tumor necrosis factor-alpha.

Preclinical studies have indicated that exercise enhances the levels of pivotal antioxidant enzymes, anti-inflammatory cytokines (including IL-10), and anti-apoptotic proteins (including Bcl-2) within intestinal lymphocytes, concurrently diminishing pro-inflammatory cytokines (TNF-α and IL-17) and pro-apoptotic proteins (caspase 3 and 7). This culminates in a comprehensive attenuation of gut inflammation [206, 207]. In mice, voluntary wheel-running has been shown to modulate immune functionality by diminishing the abundance and diversity of *Turicibacter* in fecal specimens and the cecum [208]. Likewise, individuals with sedentary habits who actively engage in high-intensity interval sprints or moderately intense physical exercise exhibited reductions in both systemic and intestinal concentrations of the pro-inflammatory mediators TNF-α and LPS-binding protein [209]. Transgenic APP/PS1 AD mice underwent a treadmill exercise regimen for over 20 weeks, resulting in enhanced spatial memory and elevation in the Clostridia and *Roseburia* populations, alongside a reduced prevalence of *Prevotella*, *Bacteriodes fragilis* and other members of the genus, and *L. johnsonii* [210]. Another study indicated that APP/PS1 transgenic AD mice that underwent a regimen of 12 weeks of treadmill exercise exhibited enhancements in spatial memory, suppression of Aβ pathology, increased gut microbial diversity, and mitigation of neuroinflammation within the brain. Notably, exercise decreased pathogenic bacterial populations such as intestinal *Allobaculum*, increased beneficial probiotic bacteria such as *Akkermansia*, elevated the levels of proteins integral to the intestine-brain barrier, and reduced LPS displacement [211]. Jin et al. [212] documented that a 20-week regimen of forced treadmill exercise induced notable symbiotic alterations in the gut microbiota, demonstrated by an increase in *A. muciniphila* and a decrease in *Bacteroides* spp., alongside an enhancement in the expression of BBB-associated proteins and mitigation of cognitive deficits resembling AD, as well as a deceleration in the progression of neuropathological changes (Fig. 5).

Nonetheless, clinical investigations in this domain are scarce, and knowledge about the mechanisms underlying the effects of exercise on gut microbiota derives predominantly from animal studies. The first longitudinal study aiming to assess the repercussions of exercise on the human gastrointestinal microbiota was published only a few years ago and revealed the compositional and functional alteration of endurance training on gut microbiota and SCFAs, particularly among lean subjects, who exhibited a reduction in *Bacteroides* and an increase in *Faecalibacterium* and *Lachnospira*, followed by an increase in fecal SCFAs [205].

Antibiotic interventions that killed the intestinal microbiota simultaneously delayed the growth of neuronal cells within the hippocampal regions of mice. When considered independently, both the administration of probiotics and aerobic exercise ameliorated the reduction in neurogenesis and cognitive capabilities in mice subjected to antibiotic treatment. Nevertheless, while the extent to which *Bifidobacterium* facilitates the effects of exercise on cerebral neurogenesis and cognitive performance remains uncertain [213], exercise’s ability to influence the microbiota and the ensuing gut-brain-related pathways may have crucial implications for the development of approaches to treat NDDs (Fig. 5). Studies on the neuroprotective effects of exercise in gut-microbiota modulation are presented in Table 5.

**Table 5:** Potential therapeutic approaches are targeting the exercise to treat NDDs.

Exercise type	Impact on microbiota	Therapeutic effects	References
Voluntary wheel-running exercise	Modulation of the gut microbiota and enhancements in gut morphology	Increased health benefits and regulates inflammatory and anti-inflammatory response profiles	[205]
Freewheel exercise training and voluntary wheel-running exercise	Attenuating gut inflammation	Enhances the levels of antioxidant enzymes, anti-inflammatory cytokines IL-10, and anti-apoptotic proteins Bcl-2 by inhibiting TNF-α and IL-17 expression	[206-207]
Voluntary wheel-running exercise, moderately intense physical exercise, and treadmill exercise	Decreased abundance and diversity of *Turicibacter*, reduced systemic inflammation, and elevation in the Clostridia and *Roseburia* populations	Modulate immune function and enhance spatial memory function	[208-210]
Treadmill exercise	Increased gut microbial diversity and increased beneficial probiotic bacteria	Enhancements in spatial memory, suppression of Aβ pathology, and reduced neuroinflammation	[211]
Forced treadmill exercise	Alterations in the gut microbiota and modulates gut microbial metabolites	Increased expression of BBB-associated proteins, mitigation of cognitive deficits, and changes in the neuropathological progression	[212]
Aerobic exercise	Exercise’s ability to stimulate gut-brain-related pathways	Increased neurogenesis expression and cognitive performance	[213]

Aβ: amyloid beta, BBB: blood-brain barrier, Bcl2: B-cell lymphoma 2, IL-10: interleukin-10, IL-17: interleukin-17, TNF-α: Tumor necrosis factor-alpha.

### Future Directions

Shifts in the gut microbiota or disturbances in the microbiota-gut-brain axis can notably influence brain function, both directly and indirectly. Emerging evidence suggests that both brain-resident and gut-resident immune cells play a pivotal role in regulating communication within the microbiota-gut-brain axis. The gut microbiota exerts substantial influence over the intestinal immune system’s maturation and functionality while the innate and adaptive immune systems concurrently affect the microbiota’s composition. This bidirectional interaction fosters a symbiotic relationship with the gut microbiota, thwarting the invasion of harmful microorganisms against the host’s defensive mechanisms. Additionally, the microbiota can modulate brain microglia and astrocytes, which are integral to neurophysiological processes, encompassing neural development, neurotransmission, intestinal epithelial cell function, and activation of the CNS immune responses.

The impact of these microbial interactions on immune responses bears crucial implications for brain inflammation, neural injury, behavioral outcomes, neuroplasticity, and neurogenesis dysfunction. The gut microbiota is increasingly correlated with manifestations of various neuroinflammatory conditions and NDDs. This interrelationship facilitates a robust communication network between the CNS and the immune system, serving as vital cellular mediators throughout the gut-brain axis. Disruption of these intricate and dynamic interactions may result in substantial repercussions for the host’s health. Various innovative approaches have been employed to explore the role of the microbiota-gut-brain axis in immune-related NDDs, such as GF mice models, probiotics, antibiotics, infection studies, exercise, natural drugs, and fecal transplantation experiments.

Despite the recent burgeoning research on the microbiota-gut-brain axis, the methodologies for elucidating the direct influences of the gut microbiota on the brain remain somewhat constrained. For instance, the complexities of the BBB should be considered when investigating the reciprocal communication between the gut microbiota and the brain. To advance beyond mere correlational approaches, cutting-edge technologies are being crafted to reveal and confirm biological mechanisms of action while paving the way for novel treatments for NDDs. An enhanced understanding of the microbiota-gut-brain axis may enable therapeutic strategies to improve cognitive functionality in individuals suffering from NDDs. Consequently, a prospective therapeutic approach centered on gut microbiota promises to become a revolutionary paradigm in managing NDDs.

## Conclusions

A growing body of clinical and preclinical research has provided evidence of the interconnection relationship between gut microbiota and the brain, particularly within NDDs. The microbiota-gut-brain axis is increasingly recognized as a crucial player in the pathophysiology of NDD conditions, influencing host health through complex interactions involving the nervous, immune, and metabolic systems. A stable and balanced gut microbiota is essential for maintaining health in a homeostatic gastrointestinal environment. However, dysbiosis—characterized by microbial translocation, compositional shifts, and the subsequent release and accumulation of neurotoxic metabolites—can significantly contribute to the initiation and progression of neurodegeneration. The gut microbiome exerts its influence through the modulation of neurological and immunological signaling pathways and is important to the biosynthesis of various neurotransmitters. These interactions are pivotal in mediating neuroinflammatory responses and neurodegenerative processes associated with NDDs. The gut microbiota plays a crucial role in developing the CNS during early life and in the pathophysiology of aging-related frailty and increased barrier permeability. Dysbiosis, immune dysfunction, and gut inflammation stemming from factors such as diet, pharmacological agents, aging, and lifestyle modifications can disrupt the gut-brain axis, potentially leading to chronic systemic inflammation. This immune dysregulation is characterized by an elevated secretion of metabolic imbalances, pro-inflammatory cytokines, and stimulated permeability of both the BBB and the gastrointestinal tract, which may ultimately provoke neuroinflammation and contribute to the onset of various NDDs. Conversely, gut microbiota exhibit multiple benefits. Modulating gut microbiota composition, regulating the immune system, and increasing or appropriate concentrations of beneficial metabolites can significantly mitigate systemic and neuroinflammation, promote repair mechanisms for barrier integrity, and potentially slow disease progression. Thus, the interactions between gut microbiota and the host's immune and nervous systems underscore a complex interplay with health and disease management implications. Therefore, our review highlighted preclinical and human studies on the targeted therapeutic modulation of the microbiota-gut-brain axis. We explore the use of probiotics, prebiotics, FMT, natural drugs, and exercise interventions as potential therapeutic targets for managing NDDs. So far, high-quality preclinical studies and several randomized controlled trials have provided evidence supporting the efficacy of therapies targeting the microbiota-gut-brain axis. Additional clinical candidates aimed at modulating the gut-brain axis to address NDDs are anticipated to advance.

## Limitations

The gut microbiota significantly impacts the neuropathogenesis of NDDs through its effects on the gut-brain axis, both directly and indirectly. Despite its importance, the molecular mechanisms underpinning these interactions and potential therapeutic strategies for NDDs remain poorly elucidated. This paper summarizes current knowledge regarding the microbiota's role in the development and progression of NDDs, focusing on the interplay between microbiota and gut-brain signaling and the associations between gut microbiota and neuro-immune responses. Moreover, we discuss emerging therapeutic approaches that hold promise for treatment paradigms in NDDs. However, the current study has several limitations. Preclinical and clinical research is needed to identify the roles of specific microbiota and microbiota-derived metabolites and their impact on brain pathology. Understanding the molecular mechanisms through which certain microbiota influence the brain is crucial. Further investigation is necessary to determine which specific microbiota, their metabolites, and immune cells interact, along with the receptors and cytokines involved in this communication. Additionally, it is important to explore how neurotransmitters produced or modulated by the gut affect signaling in the ENS and the gut-brain axis, both with and without external factors such as dietary supplementation with probiotics, prebiotics, and medicinal herbs. The gut microbiome represents a promising avenue for understanding and treating NDDs. Future research should focus on the in-depth mechanisms by which microbiota interact with the immune system and impact the gut-brain axis. Additionally, effective strategies for maintaining and restoring the homeostasis of the gut microbiome ecosystem need to be identified.
